# Avoiding Drug Resistance by Substrate Envelope-Guided Design: Toward Potent and Robust HCV NS3/4A Protease Inhibitors

**DOI:** 10.1128/mBio.00172-20

**Published:** 2020-03-31

**Authors:** Ashley N. Matthew, Jacqueto Zephyr, Desaboini Nageswara Rao, Mina Henes, Wasih Kamran, Klajdi Kosovrasti, Adam K. Hedger, Gordon J. Lockbaum, Jennifer Timm, Akbar Ali, Nese Kurt Yilmaz, Celia A. Schiffer

**Affiliations:** a Department of Biochemistry and Molecular Pharmacology, University of Massachusetts Medical School, Worcester, Massachusetts, USA; Duke University School of Medicine

**Keywords:** X-ray crystallography, drug design, drug resistance mechanisms, hepatitis C virus, structural biology

## Abstract

Hepatitis C virus (HCV) infects millions of people worldwide, causing chronic liver disease that can lead to cirrhosis, hepatocellular carcinoma, and liver transplant. In the last several years, the advent of direct-acting antivirals, including NS3/4A protease inhibitors (PIs), has remarkably improved treatment outcomes of HCV-infected patients. However, selection of resistance-associated substitutions and polymorphisms among genotypes can lead to drug resistance and in some cases treatment failure. A proactive strategy to combat resistance is to constrain PIs within evolutionarily conserved regions in the protease active site. Designing PIs using the substrate envelope is a rational strategy to decrease the susceptibility to resistance by using the constraints of substrate recognition. We successfully designed two series of HCV NS3/4A PIs to leverage unexploited areas in the substrate envelope to improve potency, specifically against resistance-associated substitutions at D168. Our design strategy achieved better resistance profiles over both the FDA-approved NS3/4A PI grazoprevir and the parent compound against the clinically relevant D168A substitution. Crystallographic structural analysis and inhibition assays confirmed that optimally filling the substrate envelope is critical to improve inhibitor potency while avoiding resistance. Specifically, inhibitors that enhanced hydrophobic packing in the S4 pocket and avoided an energetically frustrated pocket performed the best. Thus, the HCV substrate envelope proved to be a powerful tool to design robust PIs, offering a strategy that can be translated to other targets for rational design of inhibitors with improved potency and resistance profiles.

## Abstract (precis)

Despite significant progress, hepatitis C virus (HCV) continues to be a major health problem with millions of people infected worldwide and thousands dying annually due to resulting complications. Recent antiviral combinations can achieve >95% cure, but late diagnosis, low access to treatment, and treatment failure due to drug resistance continue to be roadblocks against eradication of the virus. We report the rational design of two series of HCV NS3/4A protease inhibitors with improved resistance profiles by exploiting evolutionarily constrained regions of the active site using the substrate envelope model. Optimally filling the S4 pocket is critical to avoid resistance and improve potency. Our results provide drug design strategies to avoid resistance that are applicable to other quickly evolving viral drug targets.

## INTRODUCTION

Hepatitis C virus (HCV) is estimated to chronically infect over 71 million people worldwide. The clinical sequelae of HCV infection include chronic liver disease, cirrhosis from prolonged inflammation, and hepatocellular carcinoma (1). Combination therapies with direct-acting antivirals (DAAs) against essential viral proteins NS3/4A, NS5A, and NS5B have significantly improved treatment options and outcomes (2–5) with cure rates of ∼95% for treatment-naive patients (6–12). However, even the most recent DAA combinations, still in 2019, fail to cure some patients (4, 5, 13, 14). Especially for DAA-experienced patients, baseline polymorphisms among diverse genotypes and preexisting resistance-associated substitutions (RASs) negatively impact treatment outcomes (3–5, 14, 15). Treatment failure is highly associated with RASs in the therapeutic target (4, 5, 14–19). With the WHO goal to increase treatment from 13% (2016) to 80% (2030) of the 71 million infected globally (1, 20), even a small failure rate will result in many HCV-infected patients failing therapy due to drug resistance (3, 14–19, 21, 22).

The NS3/4A protease is an excellent target for developing DAAs against HCV, and protease inhibitors have been a key component of most combination therapies. This essential protease cleaves the HCV polyprotein into functional units necessary for viral replication and maturation (23). Currently three noncovalent FDA-approved protease inhibitors (PIs) are in clinical use for the treatment of HCV: grazoprevir (24), glecaprevir (25), and voxilaprevir (26). All HCV PIs have large heterocyclic P2 moieties that significantly improve potency (27). Our high-resolution crystal structures revealed how the identity and binding mode of the P2 moiety strongly influence the inhibitor resistance profile: the P2 moiety of each PI contacts, to various extents, residues Arg155, Ala156, and Asp168 (28) where the most common RASs occur. Notably, residue 168 has emerged as a key position where substitutions can cause detrimental potency loss and resistance (28, 29). Structurally Asp168 is a critical residue that contributes to an active-site electrostatic network necessary for efficient inhibitor binding. Disruption of this network underlies the mechanism of resistance due to substitutions at Arg155 or Asp168 (28, 30).

More recent PIs, starting with grazoprevir, largely thwart susceptibility to RASs at Arg155 as their P2 quinoxaline moiety stacks against two residues of the invariant catalytic triad (His57 and Asp81) and minimizes contact with variable residues (28, 31). Mutation of the catalytic triad is not possible while retaining activity, thus decreasing the likelihood of viable resistance. Accordingly, recent PIs are less susceptible to single substitutions at Asp168, but they are still susceptible to double substitutions that include changes at Asp168, as well as changes at Ala156 due to the macrocyclization of P2 to P4 (P2–P4 macrocycle) (25). The D168Q polymorphism has rendered HCV genotype 3 “naturally resistant” to most PIs (32), and Asp/Gln168 mutations have emerged in nearly all patients who fail therapy with a PI-containing regimen (15, 33). Thus, exploring alternative scaffolds and modifications to current PIs to improve potency against Asp168 substitutions can provide more robust PIs with pan-genotypic activity, decreasing incidences of treatment failure due to drug resistance.

Rational design of inhibitor modifications to avoid resistance greatly benefits from elucidation of the structural mechanisms underlying drug resistance. Drug resistance occurs when the balance between substrate recognition and cleavage is favored over inhibitor binding. The substrate envelope defines the consensus volume necessary for NS3/4A protease to recognize the viral and host substrate sequences (34), and RASs occur where inhibitors protrude from the substrate envelope and contact residues of the enzyme that are unessential for substrate recognition (28). While protrusion beyond the substrate envelope at the P2 position on the inhibitor scaffold is unavoidable without compromising potency, leveraging evolutionarily constrained residues can circumvent resistance. The P2–P4 macrocycle of grazoprevir improves inhibitor potency by restricting conformational degrees of freedom, but the macrocycle itself protrudes from the substrate envelope and contacts nonevolutionarily constrained residues. Because of the P2–P4 macrocyle, grazoprevir is highly susceptible to A156T and moderately susceptible to substitutions at Asp168 (31). Even with vulnerability to these key RASs, given the relatively improved resistance profile and potency of grazoprevir over previous PIs, the P2–P4 macrocyclic scaffold has been used in the development of the latest generation of structurally similar inhibitors glecaprevir and voxilaprevir.

**FIG 1:**
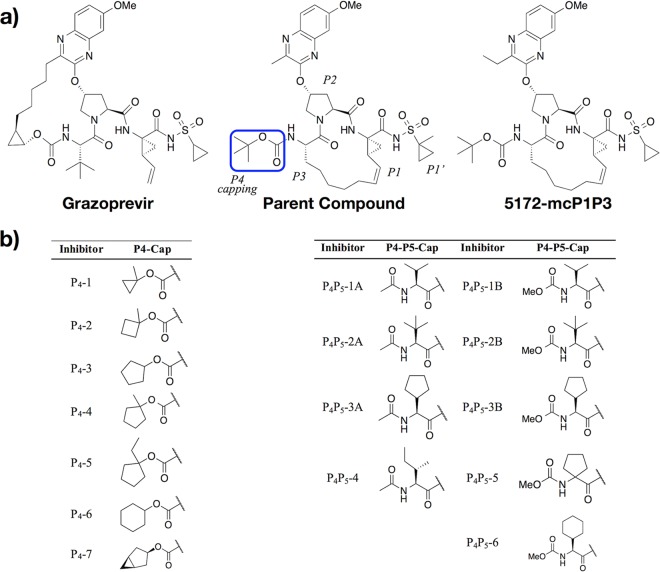
Chemical structures of designed HCV NS3/4A protease inhibitors. (a) Grazoprevir (MK-5172) is an FDA-approved PI. Change of the macrocycle location (5172-mcP1P3) and optimization of the P2 quinoxaline moiety led to the parent compound (35) modified in this study. The canonical nomenclature for drug moiety positioning and the P4 moiety altered are indicated. (b) The inhibitors designed based on the parent compound (i) to optimally fill the S4 pocket by modifying the P4 capping group (P_4_-1 to P_4_-7) and (ii) to extend into the substrate envelope by incorporating a P4 moiety with a P5 capping group (P_4_P_5_-1A to P_4_P_5_-6).

To overcome the vulnerability caused by the P2–P4 macrocycle of grazoprevir, we replaced it with a P1–P3 macrocycle previously used in danoprevir (24) to design inhibitors that can avoid resistance while retaining potency. The resulting inhibitor, 5172-mcP1P3, was less susceptible to single-site RASs, particularly A156T (29), and the crystal structures validated that the binding mode of the P2 quinoxaline moiety stacking against catalytic residues was retained (31). Further optimization by modifications at the 3-position of the P2 quinoxaline moiety to decrease interactions with the S2 subsite residues Arg155 and Ala 156 revealed that compounds with a smaller methyl group at this position retains better activity against resistant variants (35). The resulting inhibitor (Fig. 1) with the optimized P2 quinoxaline achieved an improved resistance profile and avoided susceptibility to RASs. While this was a key proof of principle that fitting within the substrate envelope is critical to avoiding susceptibility to resistance, our objective is to further explore the strategy of rationally designing inhibitors guided by the substrate envelope to improve potency and resistance profile.

The current study shows that substrate envelope-guided design achieves HCV protease inhibitors with better potency and resistance profiles. This design strategy involves decreasing interactions with variable residues that mutate to confer resistance (while keeping interactions with the invariable catalytic triad) and optimally filling the active-site pockets. We rationally designed HCV protease inhibitors that retain potency while fitting within the substrate envelope to avoid resistance, particularly against D168A, which confers high-level resistance to current HCV PIs (15, 33). Starting with the grazoprevir analog bearing a P1–P3 macrocycle and optimized P2 quinoxaline moiety, a series of inhibitors were designed and synthesized with modifications and extensions in the P4 direction proximal to D168. HCV PIs mainly span the positions of P1′ to P4, while the design here leverages a conserved region of the substrate envelope (36) that is virtually untapped by the current inhibitors. We incorporated two sets of modifications, either modifying the P4 capping (P_4_ series) or including a P4 moiety mimicking substrate interactions and extending into the P5 position (P_4_P_5_ series). A total of 16 new inhibitors were designed and synthesized to systematically explore the size and shape of the P4 moiety, potency was measured against the wild-type (WT) and D168A NS3/4A proteases in enzymatic assays, and 15 cocrystal structures with select inhibitors were determined and analyzed. All inhibitors designed to fit within the substrate envelope had a flatter resistance profile against D168A than the FDA-approved drug grazoprevir. Notably, the design strategy successfully yielded inhibitors with an order of magnitude better potency than that of grazoprevir and the parent compound against the D168A variant, while maintaining similar potency against WT. The crystal structures revealed that these inhibitors optimally fill the S4 pocket, gaining potency against both the genotype 1a (GT1a) WT protease and the D168A variant. Thus, substrate envelope-guided design can be successfully incorporated into the drug design process to provide inhibitors that are potent and less susceptible to resistance.

## RESULTS

### Substrate envelope-guided design of inhibitors.

The HCV NS3/4A protease substrate envelope is defined by the overlapping volume of the substrates (Fig. S1), and we have previously shown that inhibitors must fit within the envelope to have a flat profile against resistance (37–39). Relocating the macrocyle to fit within the substrate envelope in 5172-mcP1P3 (35) and the closely related parent compound (35) (Fig. 1) indeed achieved a flatter resistance profile than that of grazoprevir but unfortunately resulted in potency loss against the WT protease in enzymatic assays. Here, we succeed in both regaining this loss of potency and reducing susceptibility to D168A RAS. Starting with the parent compound, which has an optimized P2 quinoxaline, the design aimed to fill the S4 and S5 pockets under the restraints of the substrate envelope, with the goal of gaining potency and avoiding resistance. The S4 pocket is mostly nonpolar and can accommodate both hydrophobic and hydrophilic side chains in natural HCV substrates (40). However, prior structure-activity relationship (SAR) studies on macrocyclic and peptidic scaffolds show that hydrophobic moieties are associated with higher potencies (41–48). Thus, the inhibitors designed using molecular modeling had hydrophobic P4 moieties extending into the S4 pocket toward D168.

10.1128/mBio.00172-20.1 FIG S1 Substrate peptides bound at the active site of HCV NS3/4A protease. Download FIG S1, PDF file, 0.2 MB. Copyright © 2020 Matthew et al. 2020 Matthew et al. This content is distributed under the terms of the Creative Commons Attribution 4.0 International license.

**FIG 2:**
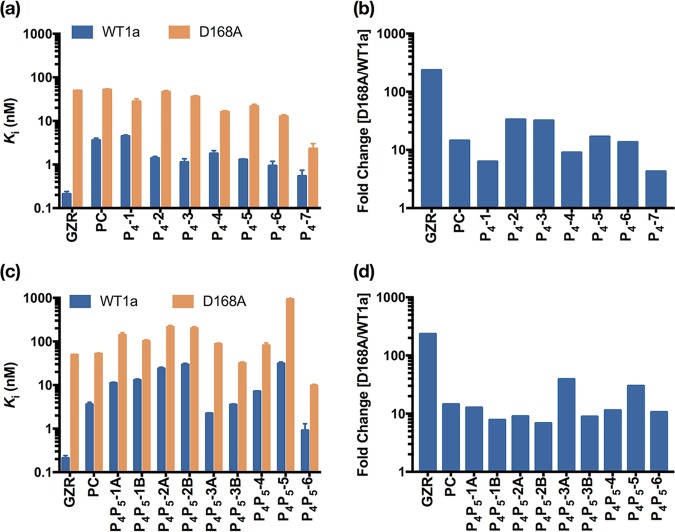
Resistance profile of HCV NS3/4A protease inhibitors against GT1a WT (WT1a) and D168A variant. (a) Enzyme inhibition constants of the P_4_-cap inhibitors against wild-type and D168A proteases, as indicated (a), and fold change of enzyme inhibitory activity against the D168A variant with respect to that of the wild-type NS3/4A protease (b). (c) Enzyme inhibition constant. (d) Fold change of the P4-P5-cap inhibitors. PC, parent compound; GZR, grazoprevir.

Two series of inhibitors were designed and synthesized, one modifying the P4 capping group to better fill the S4 pocket (P_4_ series) and the other extending into the S5 pocket with two different capping groups (P_4_P_5_ series) (Fig. 1). Specifically, P_4_ inhibitors were designed with hydrophobic P4 capping groups of increasing size to optimally fill the S4 pocket. Inhibitors in the P_4_P_5_ series aim to further tap into the unleveraged part of the substrate envelope with a hydrophobic P4 moiety and a P5 capping group. In total, we designed and synthesized 16 inhibitors, 7 with different P4 capping groups filling the S4 pocket and 9 that extend into the S5 position with either an acetamide (4 in the P_4_P_5_-A series) or a methyl carbamate (5 in the P_4_P_5_-B series) as the capping group (Fig. 1) (see Materials and Methods). For all the inhibitors, potency against HCV NS3/4A genotype 1a (GT1a) and the D168A variant was measured (Fig. 2; see Table S1 in the supplemental material), and when feasible their corresponding crystal structures were determined to elucidate the structural basis for the alterations in potency (Table S2).

10.1128/mBio.00172-20.6 TABLE S1 Inhibitory activity against wild-type (WT) GT1a HCV NS3/4A and D168A proteases in enzymatic assays. Download Table S1, PDF file, 0.1 MB. Copyright © 2020 Matthew et al. 2020 Matthew et al. This content is distributed under the terms of the Creative Commons Attribution 4.0 International license.

10.1128/mBio.00172-20.7 TABLE S2 X-ray data collection and crystallographic refinement statistics. Download Table S2, PDF file, 0.2 MB. Copyright © 2020 Matthew et al. 2020 Matthew et al. This content is distributed under the terms of the Creative Commons Attribution 4.0 International license.

### Inhibitors achieved improved potency against WT protease and RAS variant.

To optimally fill the S4 pocket, the size of the P4 capping moiety was systematically increased from 1-methylcyclopropyl to cyclohexyl and included a bicyclic capping group, bicyclo[3.1.0]hexyl, to further increase interactions in the S4 pocket. The resulting P_4_-series inhibitors ranged in potency from 4 to 0.5 nM against WT protease in enzyme inhibition assays. Compared with the parent compound, P_4_-1, the inhibitor with the smallest P4 capping group in the series, maintained the same potency against the WT (Fig. 2a and Table S1). Increasing the cyclic ring system by one carbon (P_4_-2) led to a 4-fold increase in potency against the WT. Further increase in the size of the hydrophobic P4 capping group to a cyclopentyl (P_4_-3), with addition of 1-methyl (P_4_-4) or 1-ethyl (P_4_-5), or to a cyclohexyl (P_4_-6) either maintained or slightly improved the potency further compared to that of P_4_-2 against the WT. The largest bicyclic capping group (P_4_-7) notably achieved subnanomolar potency against WT protease (0.54 ± 0.20 nM), which is comparable with the potency of grazoprevir (0.21 ± 0.03 nM) and approximately 6.5-fold more potent than that of the starting parent compound.

All of the P_4_ inhibitors were also tested against the D168A RAS and except for P_4_-2 performed better than the parent compound and grazoprevir (both ∼50 nM), ranging in potency from 2 to 36 nM. The smallest cyclic ring in P_4_-1 resulted in an ∼2-fold potency increase compared to that of the parent compound, while further increasing the size of the cyclic ring system achieved a 2.5- to 4-fold increase in potency for the cyclopentyl (P_4_-3, P_4_-4, and P_4_-5) and cyclohexyl (P_4_-6) capping groups. Incorporation of 1-methyl substituent to the cyclopentyl P4 cap (from P_4_-3 to P_4_-4) increased the potency against D168A by ∼2-fold, while a 1-ethyl substituent (P_4_-5) did not improve the potency further. The largest bicyclic capping group (P_4_-7) led to a dramatic ∼20-fold increase in potency compared to that of the parent compound and of grazoprevir against D168A, specifically retaining 2.3 ± 0.7 nM potency in contrast to that of 49.1 ± 0.6 nM for grazoprevir. Thus, the designed inhibitors succeeded in retaining nanomolar potency against the key D168A RAS variant.

Next, potency was tested for the P_4_P_5_ series of inhibitors, which were designed to fit further within the substrate envelope and extend into the S5 position. Overall, these inhibitors were less potent than the P_4_ series against both the WT (1 to 30 nM) and D168A (10 to 900 nM) protease. Inhibitors with an acetamide (P_4_P_5_-A series) versus a methyl carbamate (P_4_P_5_-B series) capping group were comparable. As in the P_4_ series, the P4 group was increased in size starting with a valine amino acid (P_4_P_5_-1A and -1B) and then increased to *tert*-leucine (P_4_P_5_-2A and- 2B) and isoleucine (P_4_P_5_-4). Larger cyclic unnatural amino acids were also incorporated in the P4 position in P_4_P_5_-3A and -3B, P_4_P_5_-5, and P_4_P_5_-6. Increasing the size of the P4 amino acid from valine to *tert-*leucine led to an ∼2-fold loss in potency against both the WT and D168A, while P_4_P_5_-4 with an isoleucine moiety was more potent than both. These inhibitors with an acyclic aliphatic P4 group were generally less potent than inhibitors containing cyclic moieties against both the WT and D168A. Generally, the P_4_P_5_ inhibitors retained a flat binding profile against D168A, losing only 8- to 13-fold potency. The cyclohexylglycine P4 amino acid in P_4_P_5_-6 yielded the most potent inhibitor in this series, comparable with the best inhibitor in the P_4_ series (P_4_-7), with potency of 0.91 ± 0.38 nM and 9.68 ± 0.64 nM against the WT and D168A variant, respectively.

### Structure determination of protease inhibitor complexes.

**FIG 3:**
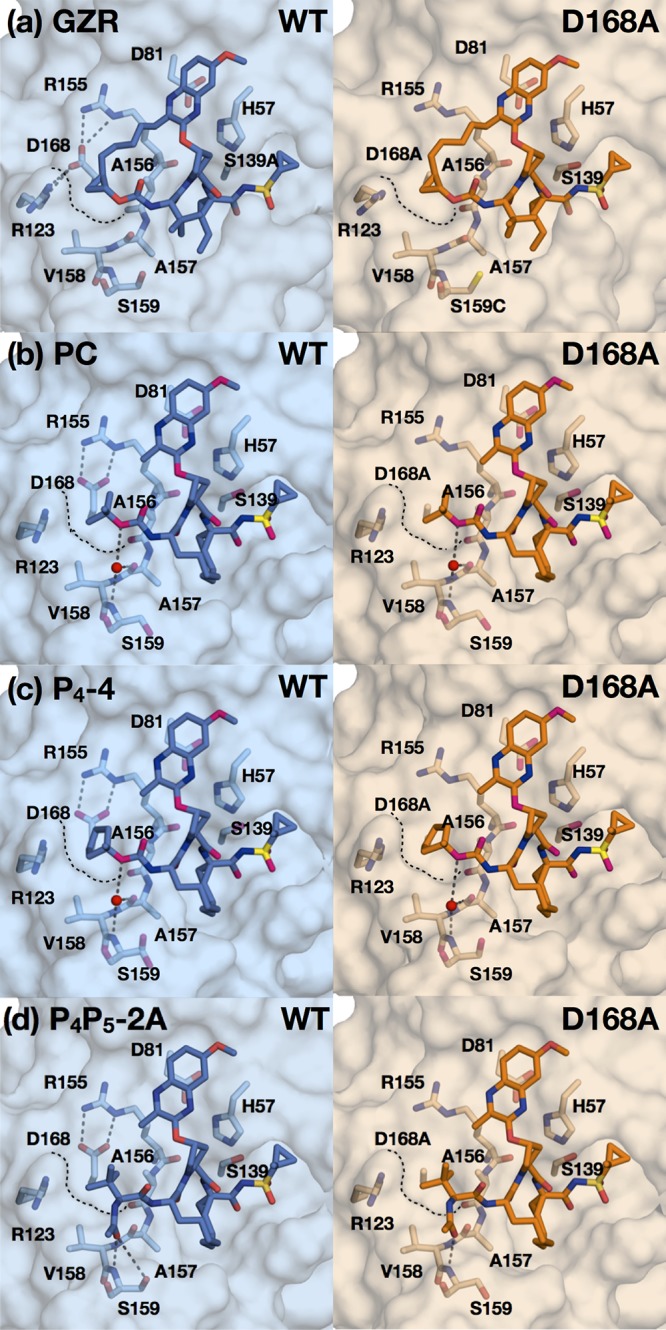
Binding of grazoprevir and designed PIs to WT and D168A protease active sites. Crystal structures of grazoprevir (GZR) (a), the parent compound (PC) (b), P_4_-4 (c), and P_4_P_5_-2A (d) bound to the wild-type and D168A proteases, as indicated. The protease active site is in surface representation, with the side chains of catalytic triad and S4 subsite residues shown as sticks. Water molecules are shown as nonbonded spheres (red), and hydrogen bonds (gray dashed lines) that stabilize S4 pocket side chains are displayed. Black dashed lines outline the surface of the S4 pocket where the D168A mutation is located.

Crystal structures of select inhibitors bound to WT and/or D168A NS3/4A protease were determined to evaluate whether the inhibitors fit within the substrate envelope as designed. A total of 15 new cocrystal structures with resolutions ranging from 1.6 to 2.1 Å were determined for this study (Table S2). Nine crystal structures of the P_4_ series included all inhibitors (except P_4_-3) bound to the D168A variant and two (P_4_-3, P_4_-4) bound to the WT protease. Six crystal structures of the P_4_P_5_ series included P_4_P_5_-2A and P_4_P_5_-2B in complex with the WT and P_4_P_5_-2A, P_4_P_5_-4, P_4_P_5_-5, and P_4_P_5_-6 with the D168A variant. All structures were analyzed in comparison with the crystal structures of grazoprevir (PDB identifier [ID] 3SUD for the WT and 3SUF for D168A) (Fig. 3a) and the parent compound (Fig. 3b) (PDB ID 5VOJ for the WT) (28, 35). As expected, the binding modes of the designed inhibitors were very similar (Fig. 3c and d). Critically, the P2 quinoxaline maintained the π-π stacking interaction with the catalytic His57 residue irrespective of modifications at the P4 and P5 positions. Alterations in binding, including hydrogen bonding interactions, occurred locally at the positions that were modified, with the P1–P3 macrocycle of the ligand relatively unchanged. The reduced potency of the inhibitors against the D168A variant, as with grazoprevir, is due to the disruption of the electrostatic network involving the Arg155 side chain as a result of the D168A substitution (Fig. 3) (28). The overall structure and binding mode of the inhibitors bound to D168A were very similar to those of the WT protease.

### Inhibitor potency and fit within the substrate envelope.

**FIG 4:**
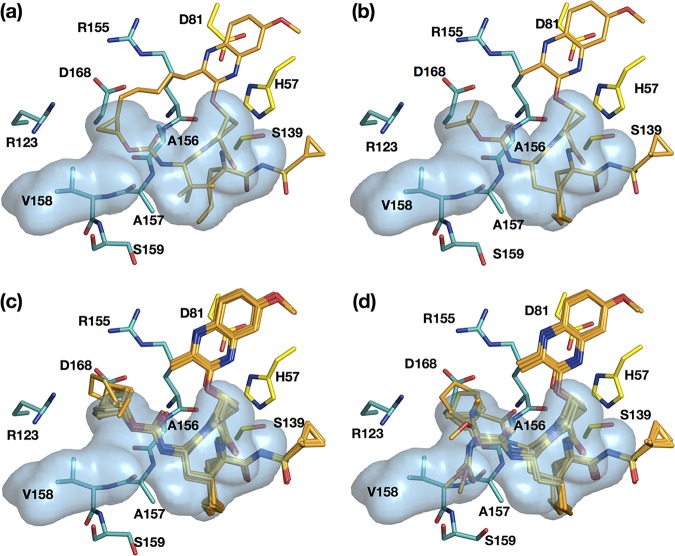
Fit of NS3/4A protease inhibitors within the substrate envelope. Inhibitors grazoprevir (a), parent compound (b), P_4_ series (P_4_-1, P_4_-2, P_4_-3, P_4_-4, P_4_-5, P_4_-6, and P_4_-7) (c), and P_4_P_5_ series (P_4_P_5_-2A, P_4_P_5_-2B, P_4_P_5_-4, P_4_P_5_-5, and P_4_P_5_-6) (d) are shown as sticks (orange) in the substrate envelope (blue). The side chains of the catalytic triad and residues surrounding the S4 pocket are shown in the substrate-bound conformations as yellow and green sticks, respectively.

The fit of the inhibitors within the substrate envelope, which was determined based on substrate-bound crystal structures (Fig. S1), was evaluated. The P2–P4 macrocycle of grazoprevir protrudes from the substrate envelope (Fig. 4a), contributing to high susceptibility to RASs proximal to the P2–P4 macrocycle and P4 capping moiety, as we previously reported (15). The parent compound, which has a P1–P3 macrocycle, fits better in the substrate envelope (Fig. 4b) (22). Inhibitors in both of the current series, P_4_ (Fig. 4c) and P_4_P_5_ (Fig. 4d), also successfully fit within the substrate envelope with two exceptions: the P4 cyclohexyl capping group of P_4_-6 and the P5 capping group of P_4_P_5_-5. Nevertheless, both series succeed in the goal of leveraging unexplored space within the substrate envelope in contrast to grazoprevir and the parent compound.

**FIG 5:**
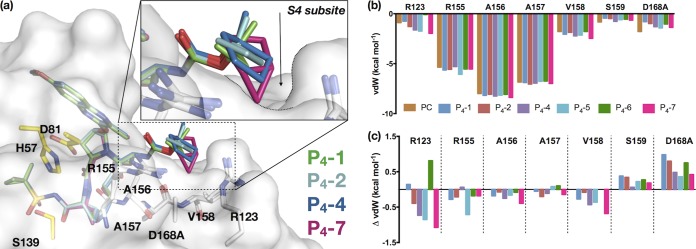
Filling the S4 subsite of the HCV NS3/4A protease active site. (a) Crystal structures of P_4_-1, P_4_-2, P_4_-4, and P_4_-7, as indicated, bound to D168A HCV protease variant. The protease active site is in surface representation, with the residues that make up the S4 pocket (white) and the catalytic triad (yellow) side chains displayed as sticks. (b) Intermolecular van der Waals (vdW) contact energies for inhibitors with residues forming the S4 pocket in the D168A crystal structures. (c) Change in vdW contacts (ΔvdW) relative to those of the parent compound (PC). Negative values indicate enhanced contacts compared to those of the parent compound.

More specifically to evaluate capping groups in the P_4_ series, the cocrystal structures bound to WT (P_4_-3), D168A (P_4_-2, P_4_-4, and P_4_-5), or both (P_4_-6 and P_4_-4) protease variants were analyzed with a focus on the S4 pocket of the substrate envelope. The small cyclopropyl ring of P_4_-1 is able to fit in the S4 pocket better than the larger ring of P_4_-2. The cyclobutyl ring of P_4_-2 is actually slightly elevated out of the pocket (Fig. 5a), similar to the structure of the parent compound (Fig. 3b), and does not have the conformational flexibility to contour the enzyme. This lack of flexibility results in an unsatisfied or “frustrated” pocket that is neither filled by the inhibitor nor has space for water to easily occupy. This structural frustration likely accounts for the weaker affinity of P_4_-2 against the D168A variant. The two inhibitors that fit the best within this pocket are P_4_-4 and P_4_-7. The P_4_-4 ring pucker and the bicyclic capping group of P_4_-7 allow for the ideal orientation toward the base of the S4 pocket (Fig. 5a). These two compounds are the most potent inhibitors against both the WT and D168 variant.

**FIG 6:**
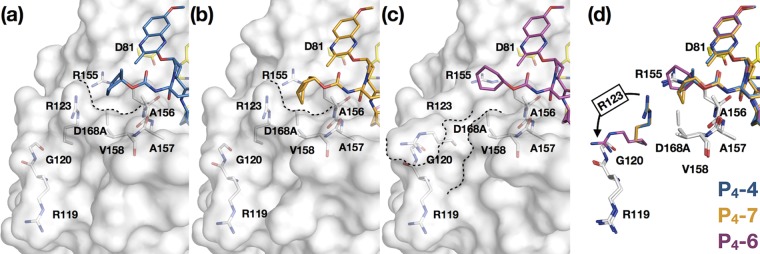
Fit of P4 capping groups and the conformation of R123 reshaping the S4 pocket of HCV NS3/4A protease. P4-4 (a), P4-7 (b), and P4-6 (c) cocrystal structures are shown with the D168A variant. The protease is in surface representation, with residues in and around the S4 pocket (white) and the catalytic triad (yellow) side chains in stick representation. The contour of the S4 pocket is outlined in dotted lines. (d) Superposition of P4-4, P4-7, and P4-6, as indicated, showing the alternate conformations of Arg123 (in respective color of the inhibitors) in the cocrystal structures.

The similarly potent P_4_-6 with the larger cyclohexyl capping group has a binding mode that differs from that of the other P_4_ inhibitors. As mentioned above, this capping group protrudes from the substrate envelope (Fig. 4c). When P_4_-6 was bound, Arg123 adopted an alternate conformation that is not observed in the other cocrystal structures with this inhibitor scaffold (Fig. 6), exposing a new groove within the protein. We have observed this alternate conformation of the Arg123 side chain in previous crystal structures of WT GT1a protease with macrocyclic and peptidomimetic inhibitors (PDB IDs 3KEE and 3SUF) and the D168A variant with danoprevir (PDB ID 1W3C). Additionally, in our structure of the D168A variant with P_4_P_5_-5, which also protrudes from the substrate envelope, we observed both conformations of Arg123.

All P_4_ inhibitors had enhanced total van der Waals (vdW) contacts compared to levels of the parent compound (Fig. S2), maintaining extensive contacts with residues 155 to 158 (Fig. 5b). The largest enhancements over the parent compound occurred within the S4 pocket, including interactions with Arg123, Arg155, and Val158 (Fig. 5c). The exception to this trend was P_4_-6 with the alternate conformation of Arg 123 (Fig. 6d). Most importantly, all of the P_4_ inhibitors had reduced vdW contacts with D168A relative to those of the parent compound, which likely underlies better potency against this RAS.

10.1128/mBio.00172-20.2 FIG S2 Total van der Waals (vdW) contact energies of (a) P_4_-cap and (b) P_4_P_5_-cap inhibitors with HCV NS3/4A D168A protease. Download FIG S2, PDF file, 0.1 MB. Copyright © 2020 Matthew et al. 2020 Matthew et al. This content is distributed under the terms of the Creative Commons Attribution 4.0 International license.

**FIG 7:**
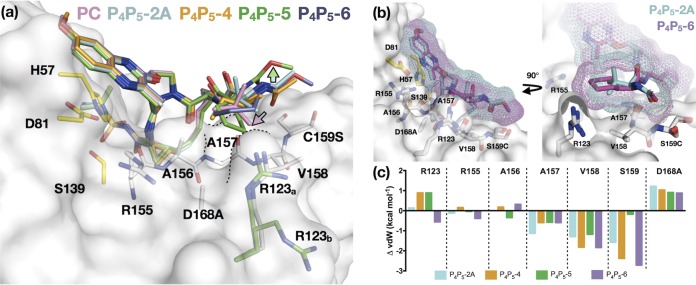
Binding mode of P_4_P_5_ inhibitors relative to that of the parent compound. (a) Superposition of cocrystal structures of the parent compound (PC), P_4_P_5_-2A, P_4_P_5_-4, P_4_P_5_-5, and P_4_P_5_-6, as indicated. The protease is in surface representation, and side chains of residues in and around the S4 pocket (white) and the catalytic triad (yellow) are displayed as sticks. The R123 can adopt two conformations (R123_a_ and R123_b_). In all of the structures, R123 is in the commonly observed conformation (white; R123_a_) except for the P_4_P_5_-5 complex (green), where both conformations are observed (green). The contour of the S4 pocket is outlined in black dotted lines. The cyan-to-pink arrow indicates the displacement of *tert*-butyl group in the parent compound relative to that in P_4_P_5_-2A, and the green arrow shows a shift of the P5 extension away from the protease surface in P_4_P_5_-5 relative to that of the other compounds. (b) Superposition of P_4_P_5_-2A and P_4_P_5_-6 bound to D168A protease. The inhibitors are displayed as sticks with a mesh surface representation of the van der Waals surface. (c) Change in vdW contacts (ΔvdW) relative to those of the parent compound (PC) for P_4_P_5_-2A, P_4_P_5_-4, P_4_P_5_-5, and P_4_P_5_-6 with S4 residues.

Within the P_4_P_5_ series, the binding modes of acetamide (P_4_P_5_-A) and methyl carbamate (P_4_P_5_-B) inhibitors were very similar (Fig. S3). The decrease in potency for the P_4_P_5_ inhibitors can largely be attributed to the P5 capping elevating the P4 group out of the S4 pocket. Therefore, as was the case for P_4_-2, the P_4_P_5_ inhibitors also create a frustrated S4 pocket (Fig. 7a). In spite of creating a frustrated pocket, the P_4_P_5_ inhibitor series demonstrate a trend similar to that of the P_4_ series, in which increasing the size of the cycloalkyl P4 group led to better potency. Importantly, not only the size but also proper orientation of the P4 group toward the S4 pocket was required for better potency. When the cycloleucine was oriented away from the pocket (P_4_P_5_-5), potency decreased, while increasing the P4 group from cyclopentylglycine (P_4_P_5_-3B) to cyclohexylglycine (P_4_P_5_-6) led to a 30-fold and 90-fold improvement against the WT and D168A, respectively. The binding and interactions of designed inhibitors in the crystal structures, particularly those of P_4_-7 and P_4_P_5_-6 (Fig. 7b and c), confirm the need for PIs to optimally fill the S4 pocket to be more potent.

10.1128/mBio.00172-20.3 FIG S3 HCV NS3/4A inhibitors in the P_4_P_5_ series with acetamide (P_4_P_5_-A) and methyl carbamate (P_4_P_5_-B) capping groups have the same binding mode. Download FIG S3, PDF file, 0.1 MB. Copyright © 2020 Matthew et al. 2020 Matthew et al. This content is distributed under the terms of the Creative Commons Attribution 4.0 International license.

## DISCUSSION

Drug resistance is a major obstacle in the design of inhibitors that remain active against rapidly evolving drug targets. As is the case with HIV, in HCV the emergence of resistance is due to low fidelity of replication, which leads to a heterogeneous viral population and selection of resistant variants in infected patients. This evolution is constrained by the requirement of the virus to replicate or by the biological function of the viral proteins. Thus, exploiting evolutionarily conserved regions in the protease active site is a rational inhibitor design strategy to decrease the probability of drug resistance. The substrate envelope model offers a guide in structure-based drug design to avoid interactions with residues that can mutate without compromising substrate processing. Protrusion of inhibitors from the substrate envelope causes vulnerability to resistance mutations. The pivotal D168A RAS in the HCV NS3/4A protease is one such mutation that impacts all of the latest-generation PIs, including grazoprevir (29, 35). In this study, we designed, synthesized, and tested inhibitors that are both potent and robust against D168A RAS and retain potency against the WT enzyme. Thus, we demonstrate that the substrate envelope strategy can successfully guide drug design to improve resistance profiles and potency.

While the most recent FDA-approved PIs (grazoprevir, voxilaprevir, and glecaprevir) have improved resistance profiles, they are still susceptible to RASs, especially at residues 156 and 168. All of these PIs have similar resistance profiles, which is not surprising considering the high similarity in their structures with a shared scaffold and P2–P4 macrocycle. Structurally, susceptibility to these RASs is due to the protrusion of the P2–P4 macrocycle from the substrate envelope and a relatively small P4 capping group constrained by the macrocycle. Moreover, the P2–P4 macrocycle limits similar SAR explorations, as well as physically constraining the P4 group. Our P1–P3 macrocylic scaffold both alleviated susceptibility to A156 RASs and enabled SAR exploration to improve potency against the WT and D168 variant. The design strategy involved introducing modifications to further fill the substrate envelope in the S4-S5 direction. By systematically increasing the size and shape of the P4 group, we found that optimally filling the S4 pocket is critical to achieving potency. Both the P_4_ and P_4_P_5_ series improved in potency over that of grazoprevir and the parent compound against the highly resistant D168A variant, and inhibitors that best filled the substrate envelope were the most potent. The most potent inhibitor from the P_4_ series, P_4_-7, has a potency similar to that of grazoprevir against WT protease but is 20-fold more potent than grazoprevir against the D168A variant. Given that the HCV genotype 3 protease contains natural polymorphisms including R123T, the PIs described here may need to be further optimized against the GT3 protease as T123 can efface the S4 pocket. Altogether, these results validated that the substrate envelope can be exploited as a strategy in rational drug design to yield potent and robust inhibitors.

In addition to avoiding resistance, with the cocrystal structures determined we also revealed the molecular basis for improved potency and the reason why some of the designed inhibitors did not retain potency. Inhibitors with relatively poor potency did not optimally fill the S4 pocket, causing a frustrated pocket that could not be filled by water, a protein side chain, or the inhibitor. This pocket was further destabilized in the D168A variant. The molecular mechanism underlying relatively low potency and resistance resembles cavity-creating mutations that destabilize protein structures (49). Unsolvated nonpolar cavities can be unfavorable for ligand binding as well (50) since they produce frustrated sites, and targeting these pockets, as we demonstrated with the S4 pocket, can significantly increase inhibitor affinity. Employing a bump-and-hole principle to increase steric complementarity between the ligand and target is common in structure-based drug design (51). Identifying pockets/cavities to target without introducing moieties vulnerable to resistance mutations is possible by a substrate envelope-guided design strategy. Thus, to improve potency and resistance profile simultaneously, we propose a substrate envelope-guided approach that optimally fills active-site pockets.

In quickly evolving drug targets, having inhibitors that bind with high potency only to the WT form of the target is not sufficient to achieving a robust drug, and strategically decreasing susceptibility to RASs within the target is necessary to avoid loss of activity due to resistance. Leveraging evolutionarily conserved regions of the target, especially the substrate-binding interactions, is critical to design such inhibitors. The substrate envelope model provides a rational and broadly applicable design strategy toward this goal for the identification of inhibitors that are more robust against drug-resistant variants.

## MATERIALS AND METHODS

### Inhibitor design and synthesis.

The compounds were computationally modeled using Maestro from Shrödinger, starting from the crystal structure of the parent compound bound to WT protease (PDB ID 5VOJ). Grazoprevir, the parent compound, and substrate envelope-designed analogs were synthesized in-house using previously reported methods (see Scheme S1 in the supplemental material). Grazoprevir was prepared according to a reported synthetic method (24). The parent compound and analogs were synthesized using our convergent reaction sequence as previously described, with minor modifications (see Text S1 for supplemental chemistry details) (29).

10.1128/mBio.00172-20.4 TEXT S1 Chemistry: synthesis and nuclear magnetic resonance/ mass spectrometry (NMR/MS) characterization of HCV NS3/4A protease inhibitors. Download Text S1, PDF file, 0.3 MB. Copyright © 2020 Matthew et al. 2020 Matthew et al. This content is distributed under the terms of the Creative Commons Attribution 4.0 International license.

10.1128/mBio.00172-20.5 SCHEME S1 Synthesis scheme of HCV NS3/4A protease inhibitors. Download Scheme S1, PDF file, 0.3 MB. Copyright © 2020 Matthew et al. 2020 Matthew et al. This content is distributed under the terms of the Creative Commons Attribution 4.0 International license.

### Expression and purification of NS3/4A constructs.

The HCV GT1a NS3/4A protease gene described in the Bristol Myers Squibb patent was synthesized by GenScript and cloned into a PET28a expression vector (52). Cys159 was mutated to a serine residue to prevent disulfide bond formation and facilitate crystallization. The D168A gene was engineered using a site-directed mutagenesis protocol from Stratagene. Protein expression and purification were carried out as previously described (28). Briefly, transformed Escherichia coli BL21(DE3) cells were grown in Tris-borate (TB) medium containing 30 μg/ml of kanamycin antibiotic at 37°C. After cultures reached an optical density at 600 nm (OD_600_) of 0.7, they were induced with 1 mM isopropyl-β-d-thiogalactopyranoside (IPTG) and harvested after 3 h of expression. Cells were pelleted by centrifugation, resuspended in resuspension buffer (RB) (50 mM phosphate buffer, 500 mM NaCl, 10% glycerol, 2 mM β-mercaptoethanol [β-ME], pH 7.5), and frozen at −80°C for storage.

Cell pellets were thawed and lysed via a cell disruptor (Microfluidics, Inc.) two times to ensure sufficient DNA shearing. Lysate was centrifuged at 19,000 rpm for 25 min at 4°C. The soluble fraction was applied to a nickel-nitrilotriacetic acid (Ni-NTA) column (Qiagen) preequilibrated with RB. The beads and soluble fraction were incubated at 4°C for 1.5 h, and the lysate was allowed to flow through. Beads were washed with RB supplemented with 20 mM imidazole and eluted with RB supplemented with 200 mM imidazole. The eluent was dialyzed overnight (molecular-weight-cutoff [MWCO] of 10 kDa) to remove the imidazole, and the His tag was simultaneously removed with thrombin treatment. The eluate was judged >90% pure by polyacrylamide gel electrophoresis, concentrated, flash frozen, and stored at −80°C.

### Correction for the inner-filter effect.

The inner-filter effect (IFE) for the NS3/4A protease substrate was determined using a previously described method (53). Briefly, fluorescence endpoint readings were taken for substrate concentrations between 0 μM and 20 μM. Afterward, free 5-carboxyfluorescein (5-FAM) fluorophore was added to a final concentration of 25 μM to each substrate concentration, and a second round of fluorescence endpoint readings was taken. The fluorescence of free 5-FAM was determined by subtracting the first fluorescence endpoint reading from the reading at the second round. IFE corrections were then calculated by dividing the free 5-FAM florescence at each substrate concentration by the free 5-FAM florescence at zero substrate.

### Determination of Michaelis-Menten (*K_m_*) constant.

*K_m_* constants for GT1 and D168A protease were previously determined (29). Briefly, a 20 μM concentration of substrate [Ac-DE-Dap(QXL520)-EE-Abu-γ-[COO]AS-C(5-FAMsp)-NH_2_] (AnaSpec) was serially diluted into assay buffer (50 mM Tris, 5% glycerol, 10 mM dithiothreitol [DTT], 0.6 mM LDAO [*N*,*N*-dimethyldodecylamine *N*-oxide], and 4% dimethyl sulfoxide), and proteolysis was initiated by rapid injection of 10 μl of protease (final concentration, 20 nM) in a reaction volume of 60 μl. The fluorescence output from the substrate cleavage product was measured kinetically using an EnVision plate reader (Perkin-Elmer) with excitation wavelength at 485 nm and emission at 530 nm. Inner-filter effect corrections were applied to the initial velocities (*V*_o_) at each substrate concentration. Graphs of *V*_o_ versus substrate concentration were globally fit to the Michaelis-Menten equation to obtain the *K_m_* value.

### Enzyme inhibition assays.

For each assay, 2 nM NS3/4A protease (GT1a and D168A) was preincubated at room temperature for 1 h with an increasing concentration of inhibitors in assay buffer (50 mM Tris, 5% glycerol, 10 mM DTT, 0.6 mM LDAO, and 4% dimethyl sulfoxide, pH 7.5). Inhibition assays were performed in nonbinding-surface 96-well black half-area plates (Corning) in a reaction volume of 60 μl. The proteolytic reaction was initiated by the injection of 5 μl of HCV NS3/4A protease substrate (AnaSpec), to a final concentration of 200 nM, and kinetically monitored using a Perkin Elmer EnVision plate reader (excitation at 485 nm; emission at 530 nm). Three independent data sets were collected for each inhibitor with each protease construct. Each inhibitor titration included at least 12 inhibitor concentration points, which were globally fit to the Morrison equation to obtain the *K_i_* value.

### Crystallization and structure determination.

Protein expression and purification were carried out as previously described (28). Briefly, the Ni-NTA-purified WT GT1a protein was thawed, concentrated to 3 mg/ml, and loaded on a HiLoad Superdex75 16/60 column equilibrated with gel filtration buffer (25 mM morpholineethanesulfonic acid [MES], 500 mM NaCl, 10% glycerol, and 2 mM DTT, pH 6.5). The protease fractions were pooled and concentrated to 25 mg/ml with an Amicon Ultra-15 10-kDa filter unit (Millipore). The concentrated samples were incubated for 1 h with 3:1 molar excess of inhibitor. Diffraction-quality crystals were obtained overnight by mixing equal volumes of concentrated protein solution with precipitant solution (20 to 26% polyethylene glycol [PEG] 3350, 0.1 M sodium MES buffer, 1 to 4% ammonium sulfate, pH 6.5) at room temperature (RT) or 15°C in 24-well VDX hanging-drop trays. Crystals were harvested, and data were collected at 100 K. Cryogenic conditions contained the precipitant solution supplemented with 15% glycerol or ethylene glycol.

X-ray diffraction data were collected in-house using our Rigaku X-ray system with a Saturn 944 detector. All data sets were processed using HKL-3000 (54). Structures were solved by molecular replacement using PHASER (55). Model building and refinement were performed using Coot (56) and PHENIX (57), respectively. The final structures were evaluated with MolProbity (58) prior to deposition in the Protein Data Bank (PDB). To limit the possibility of model bias throughout the refinement process, 5% of the data were reserved for the free-*R* value calculation (59). Structure analysis, superposition, and figure generation were done using PyMOL (60). X-ray data collection and crystallographic refinement statistics are presented in Table S1 in the supplemental material.

### Construction of HCV NS3/4A substrate envelope.

The HCV NS3/4A protease substrate envelope was computed using a method previously described (28). The HCV viral substrates representing the product complex 3-4A (residues 626 to 631 of full-length HCV PDB ID 1CU1), 4B/5A (chain D, PDB ID 3M5N), and 5A/5B (chain A, PDB ID 3M5O) were used to construct the envelope. All structures were aligned in PyMOL using the Cα atoms of protease residues 137 to 139 and 154 to 160. Following superposition of all structures, Gaussian object maps at a contour of 0.5 were generated for each cleavage product in PyMOL (28, 61). Three consensus maps were generated representing the minimum volume occupied by any two viral substrates. The four consensus maps were summed together to generate the final substrate envelope representing the shared van der Waals volume of the viral substrates.

### Structural analysis.

Superpositions were performed in PyMol using the Cα atoms of active-site residues 137 to 139 and 154 to 160 of the protease. The D168A-parent compound complex structure was used as the reference for the alignments. The van der Waals contact energies between the protease and the inhibitors were computed using a simplified Lennard-Jones potential, as described previously:

$$V(r_{\mathrm{ij}})=4\varepsilon \left[{\left(\frac{\sigma }{r_{\mathrm{ij}}}\right)}^{12}-{\left(\frac{\sigma }{r_{\mathrm{ij}}}\right)}^{6}\right]$$

where r is the distance within 6 Å between atom pairs i of the protease and j of the inhibitor, ε is the well depth, and σ is the van der Waals radius (62).

### Data availability.

Crystal structures determined in this study were deposited in the PDB under the following accession numbers: 6UE3, 6PIZ, 6PIY, 6DIT, 6DIU, 6PJ1, 6PJ0, 6PIW, 6PIV, 6DIR, 6DIV, 6DIQ, 6PJ2, 6PIX, and 6PIU.
