# Supplementary material for: Avoiding Drug Resistance by Substrate Envelope-Guided Design: Toward Potent and Robust HCV NS3/4A Protease Inhibitors
Source: mBio. 2020 Mar 31;11(2):e00172-20. doi: 10.1128/mBio.00172-20 (PMC7157764; doi:10.1128/mBio.00172-20)
Supplement: FIG S1 [file mBio.00172-20-sf001.pdf]

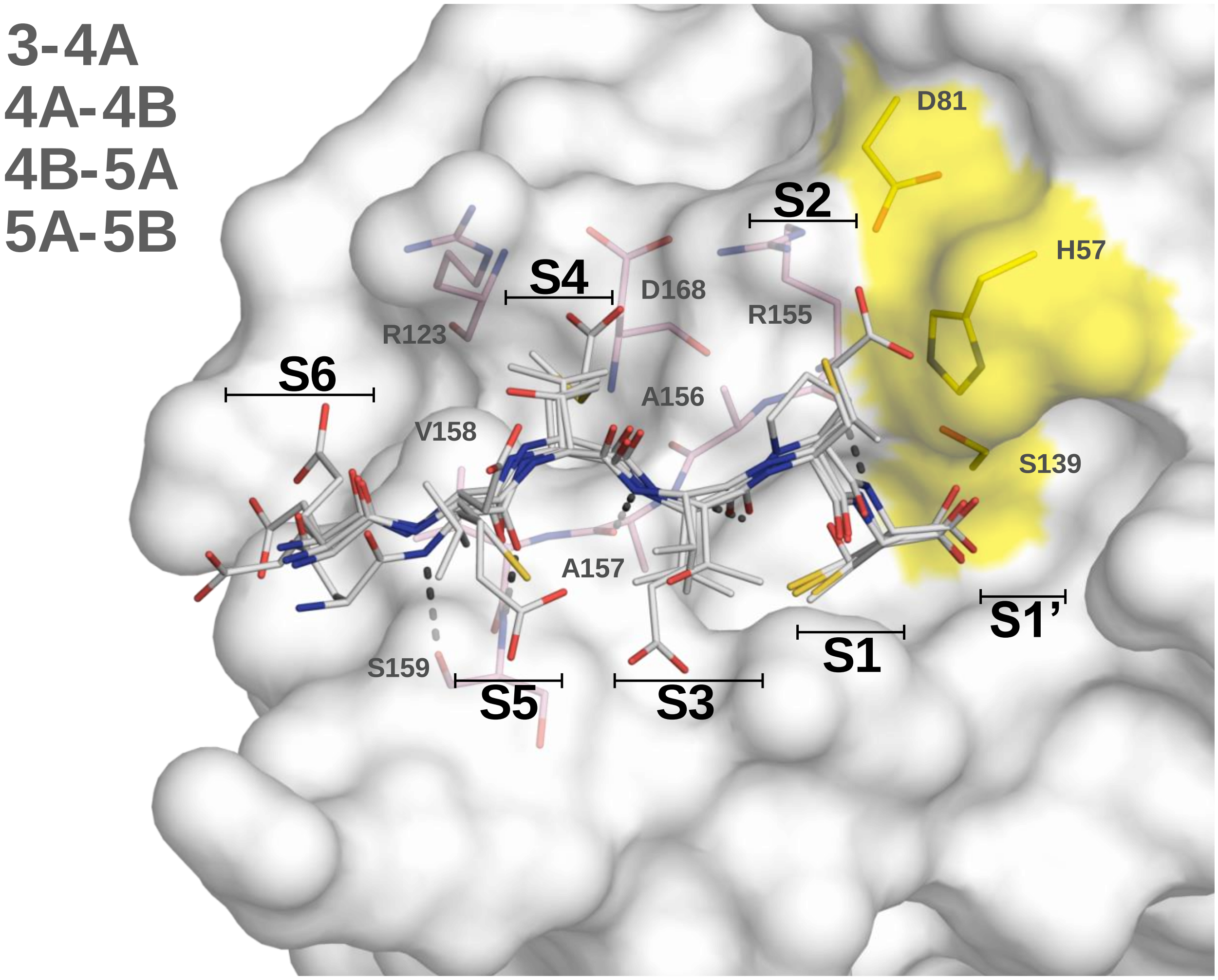

Figure S1. Substrate peptides bound at the active site of HCV NS3/4A protease. Co-crystal structures of HCV NS3/4A protease with 3-4A(1CU1), 4A-4B (3M5M), 4B-5A (3M5N), and 5A-5B (3M5O) cleavage sites. The protease is in surface representation, with the catalytic triad (yellow), Ala156, Ser159C, Arg155, Asp168, Arg123, Ala157, and Val158 (magenta) shown as stick and labeled. Backbone hydrogen bond between the substrates and the protease are shown as gray dash lines and S1'-S6 pockets are also labeled.
