# Supplementary material for: Avoiding Drug Resistance by Substrate Envelope-Guided Design: Toward Potent and Robust HCV NS3/4A Protease Inhibitors
Source: mBio. 2020 Mar 31;11(2):e00172-20. doi: 10.1128/mBio.00172-20 (PMC7157764; doi:10.1128/mBio.00172-20)
Supplement: TABLE S1 [file mBio.00172-20-st001.pdf]

Table S1. Inhibitory activity against wild-type (WT) GT1a HCV NS3/4A and D168A proteases in enzymatic assays, with fold change relative to WT indicated in parentheses. The error reported is from the global fit of at least three replicates.

Inhibitor	K_i (nM) (Fold Change)	
	GT1a WT	D168A
**Grazoprevir (GZR)**	0.21 ± 0.03 †	49.1 ± 1.6 † (234)
**5172-mcP1P3**	3.29 ± 0.52 †	82.4 ± 4.4
**Parent Compound (PC)**	3.60 ± 0.44 †	52.0 ± 2.4 § (14)
**P_4-1**	4.44 ± 0.30	27.9 ± 4.1 § (6)
**P_4-2**	1.40 ± 0.13	46.4 ± 3.4 § (33)
**P_4-3**	1.13 ± 0.22 §	36.0 ± 1.8 (32)
**P_4-4**	1.78 ± 0.30 §	16.0 ± 1.2 § (9)
**P_4-5**	1.29 ± 0.05	21.7 ± 2.3 § (17)
**P_4-6**	0.94 ± 0.25	12.7 ± 1.2 § (14)
**P_4-7**	0.54 ± 0.20	2.3 ± 0.7 § (4)
**P_4P_5-1A**	11.1 ± 0.5	141 ± 18 (13)
**P_4P_5-1B**	13.0 ± 0.8	102.0 ± 6.8 (8)
**P_4P_5-2A**	23.9 ± 1.7 §	215 ± 16 § (9)
**P_4P_5-2B**	29.5 ± 1.9 §	201 ± 15 (8)
**P_4P_5-3A**	2.23 ± 0.07	87.3 ± 4.4 (39)
**P_4P_5-3B**	3.54 ± 0.13	31.6 ± 2.1 (9)
**P_4P_5-4**	7.10 ± 0.24	81 ± 11 § (11)
**P_4P_5-5**	31.0 ± 2.74	931 ± 70 § (30)
**P_4P_5-6**	0.91 ± 0.38	9.68 ± 0.64 § (11)

§Co-crystal structures determined in this work. †Co-crystal structures previously determined by our laboratory (Soumana D.I. et al, *ACS Chem Biol*, 11(4):900, 2016, Romano K.P. et al, *Plos Pathog*, 8(7):e1002832, 2012, and (Matthew N.M. et al, *J. Med. Chem*. 60(13):5699, 2017).
