# Supplementary material for: Avoiding Drug Resistance by Substrate Envelope-Guided Design: Toward Potent and Robust HCV NS3/4A Protease Inhibitors
Source: mBio. 2020 Mar 31;11(2):e00172-20. doi: 10.1128/mBio.00172-20 (PMC7157764; doi:10.1128/mBio.00172-20)
Supplement: TABLE S2 [file mBio.00172-20-st002.pdf]

Table S2. X-ray data collection and crystallographic refinement statistics.

	**D168A PC**	**D168A P_4-1**	**D168A P_4-2**	**WT P_4-3**	**WT P_4-4**	**D168A P_4-4**	**D168A P_4-5**	**D168A P_4-6**	**D168A P_4-7**	**WT P_4P_5-2A**	**D168A P_4P_5-2A**	**WT P_4P_5-2B**	**D168A P_4P_5-4**	**D168A P_4P_5-5**	**D168A P_4P_5-6**
**PDB ID**	6UE3	6PIZ	6PIY	6DIT	6DIU	6PJ1	6PJ0	6PIW	6PIV	6DIR	6DIV	6DIQ	6PJ2	6PIX	6PIU
**Resolution (Å)**	1.56	1.89	1.86	1.79	1.87	1.89	2.05	1.90	2.14	1.75	1.83	1.58	2.10	1.87	2.28
**Space group**	$P2_12_12_1$	$P2_12_12_1$	$P2_12_12_1$	$P2_12_12_1$	$P2_12_12_1$	$P2_12_12_1$	$P2_12_12_1$	$P2_12_12_1$	$P2_12_12_1$	$P2_12_12_1$	$P2_12_12_1$	$P2_12_12_1$	$P2_12_12_1$	$P2_12_12_1$	$P2_12_12_1$
**Molecules in AU[a]**	1	1	1	1	1	1	1	1	1	1	1	1	1	1	1
**Cell dimensions:**															
**a (Å)**	55.6	54.9	55.2	55.3	55.1	54.4	55.2	54.1	55.2	55.1	55.5	55.5	54.4	54.2	54.8
**b (Å)**	58.5	58.6	58.7	58.6	59.6	58.5	58.5	58.6	58.7	59.8	58.7	58.5	59.6	58.6	59.6
**c (Å)**	59.9	59.9	59.8	59.8	58.5	60.0	59.7	59.9	60.0	58.5	60.0	59.9	58.6	59.7	58.6
**β (°)**	90	90	90	90	90	90	90	90	90	90	90	90	90	90	90
**Completeness (%)**	98.3	99.7	99.5	98.1	97.4	98.6	99.1	95.7	99.9	97.0	98.1	91.3	99.1	98.1	99.8
**Total reflections**	249313	109214	113571	118729	110776	104473	73797	104326	77445	119548	115996	250177	77900	110082	53741
**Unique reflections**	28103	15981	16808	18610	16126	15695	12209	14973	11259	19508	17556	25037	11513	15984	9233
**Average I/σ**	25.2	20.0	27.5	14.5	12.7	20..7	18.7	19.5	26.6	8.8	12.8	7.9	16.6	34.2	14.0
**Redundancy**	8.9	6.8	6.8	6.4	6.9	6.6	6.0	7.0	6.9	6.1	6.6	10	6.7	6.9	5.8
**R_{sym} (%)[b]**	4.7 (14.3)	9.5 (28.2)	7.0 (28.7)	7.1 (28.4)	6.3 (19.6)	8.5 (36.6)	9.1 (34.8)	9.5 (36.7)	7.4 (17.9)	4.3 (18.3)	7.0 (21.6)	5.7 (15.2)	11.9 (40.1)	5.7 (19.3)	11.9 (50.0)
**RMSD[c] in:**															
**Bond lengths (Å)**	0.015	0.009	0.006	0.004	0.02	0.005	0.01	0.01	0.002	0.009	0.006	0.007	0.002	0.01	0.03
**Bond angles (°)**	1.6	1.2	1.2	0.8	0.6	1.1	0.7	1.3	0.7	1.3	1.1	1.0	0.76	1.3	0.8
**R_{factor} (%)[d]**	14.4	16.0	15.8	18.2	18.3	16.4	17.7	16.9	17.4	14.6	16.1	15.1	18.2	15.2	19.3
**R_{free} (%)[e]**	17.0	19.8	17.5	22.9	23.3	20.1	21.5	20.9	20.6	19.3	19.4	18.3	21.4	19.1	23.5

[a]AU, asymmetric unit.
[b]$R_{sym} = \Sigma \mid I - \langle I\rangle \mid / \Sigma I$, where I = observed intensity, $\langle I\rangle$ = average intensity over symmetry equivalent; values in parentheses are for the highest resolution shell.
[c]RMSD, root mean square deviation.
[d]$R_{factor} = \Sigma \| F_o| - |F_c\| / \Sigma|F_o|$.
[e]R_{free} was calculated from 5% of reflections, chosen randomly, which were omitted from the refinement process.
