# Supplementary material for: Avoiding Drug Resistance by Substrate Envelope-Guided Design: Toward Potent and Robust HCV NS3/4A Protease Inhibitors
Source: mBio. 2020 Mar 31;11(2):e00172-20. doi: 10.1128/mBio.00172-20 (PMC7157764; doi:10.1128/mBio.00172-20)
Supplement: FIG S2 [file mBio.00172-20-sf002.pdf]

(a)

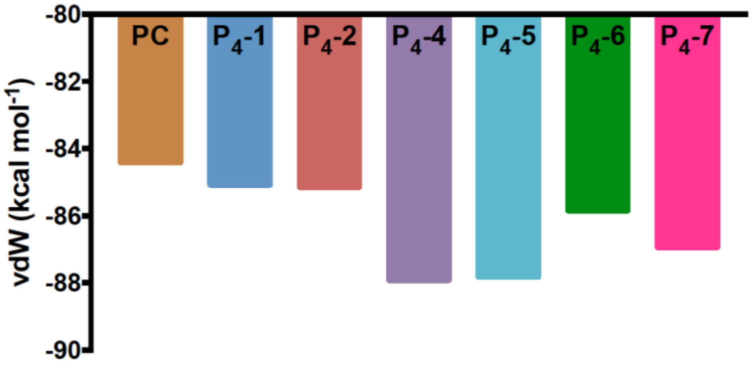

(b)

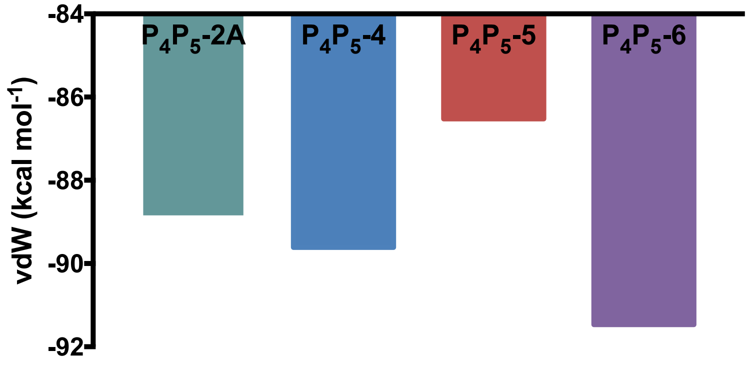

Figure S2. Total van der Waals (vdW) contact energies of (a) P_4-cap and (b) P_4P_5-cap inhibitors with HCV NS3/4A D168A protease.
