# Supplementary material for: Avoiding Drug Resistance by Substrate Envelope-Guided Design: Toward Potent and Robust HCV NS3/4A Protease Inhibitors
Source: mBio. 2020 Mar 31;11(2):e00172-20. doi: 10.1128/mBio.00172-20 (PMC7157764; doi:10.1128/mBio.00172-20)
Supplement: FIG S3 [file mBio.00172-20-sf003.pdf]

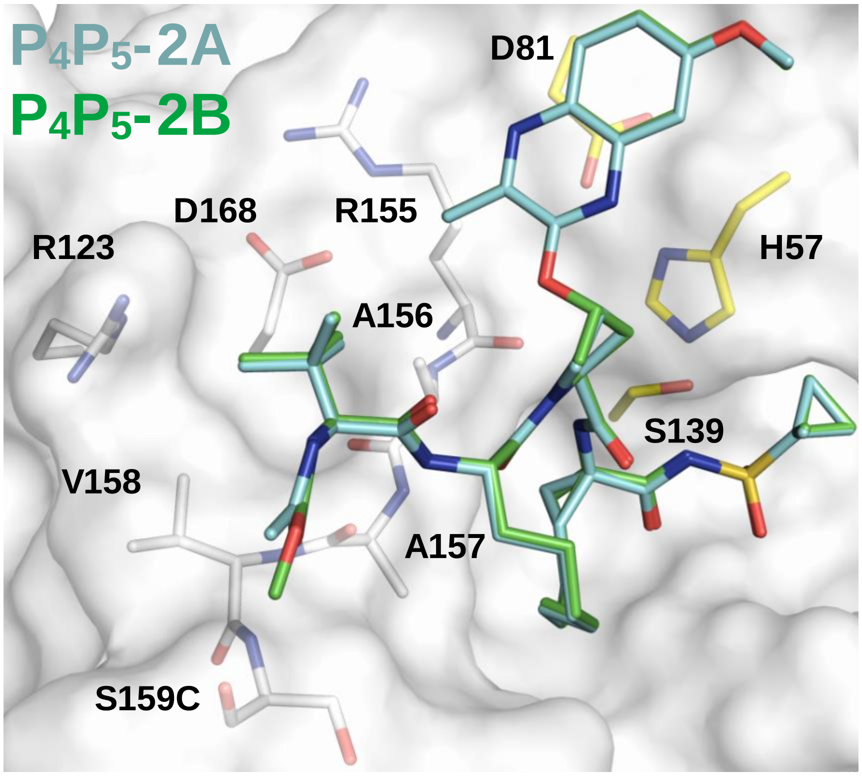

Figure S3. HCV NS3/4A inhibitors in the P_4P_5 series with acetamide (P_4P_5 A) and methyl carbamate (P_4P_5 B) capping groups have the same binding mode. Superposition of P_4P_5-2A (cyan) and P_4P_5-2B (green) in co-crystal structure with WT1a protease. The protease is in surface representation, with the side chains of residues around the S4 pocket (white) and the catalytic triad (yellow) displayed as sticks.
