# Supplementary material for: Avoiding Drug Resistance by Substrate Envelope-Guided Design: Toward Potent and Robust HCV NS3/4A Protease Inhibitors
Source: mBio. 2020 Mar 31;11(2):e00172-20. doi: 10.1128/mBio.00172-20 (PMC7157764; doi:10.1128/mBio.00172-20)
Supplement: TEXT S1 [file mBio.00172-20-s0001.pdf]

CHEMISTRY

General. No unexpected or unusually high safety hazards were encountered. All reactions were performed in oven-dried round bottomed flasks fitted with rubber septa under argon atmosphere, unless otherwise noted. All reagents and solvents, including anhydrous solvents, were purchased from commercial sources and used as received. Flash column chromatography was performed on an ISCO CombiFlash instrument using RediSep Gold columns. Thin-layer chromatography (TLC) was performed using silica gel (60 F-254) coated aluminum plates (EMD Millipore), and spots were visualized by exposure to ultraviolet light (UV), exposure to iodine adsorbed on silica gel, and/ or exposure to an acidic solution of *p*-anisaldehyde followed by brief heating. ^{1}H NMR and ^{13}C NMR spectra were acquired on a Bruker Avance III HD 500 MHz NMR instrument. Chemical shifts are reported in ppm (δ scale) with the residual solvent signal used as reference and coupling constant (*J*) values are reported in hertz (Hz). Data are presented as follows: chemical shift, multiplicity (s = singlet, d = doublet, dd = doublet of doublet, t = triplet, q = quartet, m = multiplet, br s = broad singlet), coupling constant in Hz, and integration. High-resolution mass spectra (HRMS) were recorded on a Thermo Scientific Orbitrap Velos Pro mass spectrometer coupled with a Thermo Scientific Accela 1250 UPLC and an autosampler using electrospray ionization (ESI) in the positive mode.

1-(*Tert*-Butyl) 2-methyl (2*S*,4*R*)-4-((7-methoxy-3-methylquinoxalin-2-yl)oxy)pyrrolidine-1,2-dicarboxylate (3).

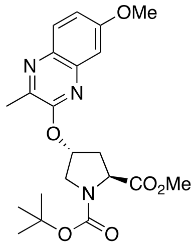

A solution of 7-methoxy-3-methylquinoxalin-2(1*H*)-one **1** (6.2 g, 32.6 mmol) in anhydrous NMP (100 mL) was treated with Cs_2CO_3 (16.0 g, 49.0 mmol). After stirring the reaction mixture at room temperature for 15 min, activated *cis*-hydroxyproline derivative **2** (14.0 g, 30.2 mmol) was added in one portion. The reaction mixture was heated to 55 °C, stirred for 4 h, and then another portion of activated *cis*-hydroxyproline **2** (1.0 g, 2.15 mmol) was added. The resulting reaction mixture was stirred at 55 °C for additional 2 h, cooled to room temperature, quenched with aqueous 1 N HCl solution (250 mL), and extracted with EtOAc (400 mL). The organic fraction was washed successively with saturated aqueous $NaHCO_3$ and NaCl (250 mL each), dried (Na_2SO_4), filtered, and evaporated under reduced pressure. The residue was purified by flash column chromatography (RediSep Gold column, 2 × 80 g, gradient elution with 0–60% EtOAc/hexanes) to provide **3** (10.0 g, 74%) as a white foamy solid. **^{1}H NMR** (500 MHz, $CDCl_3$) (mixture of rotamers, major rotamer) δ 7.80 (d, *J* = 9.0 Hz, 1 H), 7.17 (dd, *J* = 9.0, 3.0 Hz, 1 H), 7.11 (d, *J* = 2.5 Hz, 1 H), 5.71 (br s, 1 H), 4.48 (t, *J* = 8.0 Hz, 1 H), 3.99–3.91 (m, 4 H), 3.87 (d, *J* = 12.5 Hz, 1H), 3.78 (s, 3 H), 2.67–2.58 (m, 1 H), 2.56 (s, 3 H), 2.43–2.37 (m, 1 H), 1.43 (s, 9 H) ppm; **^{13}C NMR** (125 MHz, $CDCl_3$) δ 173.36, 160.24, 155.51, 153.81, 144.60, 141.04, 134.22, 128.95, 118.63, 105.95, 80.54, 73.59, 58.20, 55.68, 52.48, 52.20, 36.70, 28.26, 19.93 ppm; **HRMS** (ESI) *m/z*: $[M + H]^+$ calcd for $C_{21}H_{28}N_3O_6$, 418.1973; found 418.1976.

Methyl (2*S*,4*R*)-1-((*S*)-2-((*tert*-butoxycarbonyl)amino)non-8-enoyl)-4-((7-methoxy-3-methylquinoxalin-2-yl)oxy)pyrrolidine-2-carboxylate (6).

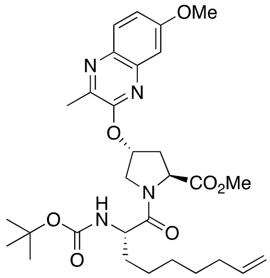

A solution of ester **3** (10.0 g, 24.0 mmol) in anhydrous CH_2Cl_2 (50 mL) was treated with a solution of 4 N HCl in 1,4-dioxane (100 mL). After stirring the reaction mixture at room temperature for 3 h, solvents were evaporated under reduced pressure, and the residue was dried under high vacuum. The pale-yellow solid was triturated with diethyl ether (3 × 25 mL) and dried under high vacuum to yield the amine salt **4** (8.5 g, 100%) as an off-white powder.

A mixture of amine salt **4** (8.5 g, 24.0 mmol) and (*S*)-2-((*tert*-butoxycarbonyl)amino)non-8-enoic acid **5** (6.70 g, 24.7 mmol) in anhydrous DMF (110 mL) was treated with DIEA (19.2 mL, 110 mmol) and HATU (14.1 g, 37.1 mmol). The resulting reaction mixture was stirred at room temperature for 4 h, then diluted with EtOAc (500 mL), and washed successively with aqueous 0.5 N HCl, saturated aqueous $NaHCO_3$, and saturated aqueous NaCl (300 mL each). The organic portion was dried (Na_2SO_4), filtered, and evaporated under reduced pressure. The residue was purified by flash column chromatography (RediSep Gold column, 2 × 80 g, gradient elution with 0–60% EtOAc/hexanes) to provide **6** (10.9 g, 80%) as a white foamy solid. ^{1}**H NMR** (500 MHz, $CDCl_3$) (mixture of rotamers, major rotamer) δ 7.81 (d, *J* = 9.0 Hz, 1 H), 7.18 (dd, *J* = 9.0, 2.5 Hz, 1 H), 7.12 (d, *J* = 2.5 Hz, 1 H), 5.84–5.75 (m, 2 H), 5.21 (d, *J* = 8.5 Hz, 1 H), 5.01–4.92 (m, 2 H), 4.75 (t, *J* = 8.0 Hz, 1 H), 4.38 (q, *J* = 7.5 Hz, 1 H), 4.18 (d, *J* = 11.5 Hz, 1 H), 4.06 (dd, *J* = 12.0, 4.5 Hz, 1 H), 3.94 (s, 3 H), 3.77 (s, 3 H), 2.69–2.64 (m, 1 H), 2.54 (s, 3 H), 2.41–2.35 (m, 1 H), 2.04 (app q, *J* = 7.0 Hz, 2 H), 1.80–1.75 (m, 1 H), 1.63–1.55 (m, 1 H), 1.46–1.24 (m, 15 H) ppm; ^{13}**C NMR** (125 MHz, $CDCl_3$) δ 172.13, 171.78, 160.27, 155.40, 155.27, 144.62, 140.89, 138.96, 134.39, 129.03, 118.73, 114.35, 105.99, 79.61, 74.30, 57.97, 55.66, 52.67, 52.43, 51.83, 34.94, 33.65, 32.66, 28.91, 28.74, 28.25, 24.68, 19.87 ppm; **HRMS** (ESI) *m/z*: $[M + H]^+$ calcd for $C_{30}H_{43}N_4O_7$, 571.3126; found 571.3128.

***Tert*-Butyl ((*S*)-1-((2*S*,4*R*)-4-((7-methoxy-3-methylquinoxalin-2-yl)oxy)-2-(((1*R*,2*S*)-1-(((1-methylcyclopropyl)sulfonyl)carbamoyl)-2-vinylcyclopropyl)carbamoyl)pyrrolidin-1-yl)-1-oxonon-8-en-2-yl)carbamate (9).**

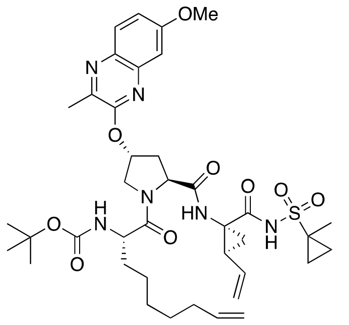

A solution of ester **6** (6.0 g, 10.5 mmol) in THF-H_2O mixture (1:1, 150 mL) was treated with LiOH.H_2O (1.55 g, 36.9 mmol). The resulting reaction mixture was stirred at room temperature for 24 h. The reaction mixture was cooled to ~5 °C, acidified to a pH of 2.0 by slow addition of aqueous 0.25 N HCl (~ 200 mL), and extracted with EtOAc (2 × 400 mL). The organic portions were washed separately with saturated aqueous NaCl (200 ml), dried (Na_2SO_4), filtered, and evaporated under reduced pressure. The gummy residue was dissolved in $CHCl_3$ (50 mL), concentrated under reduced pressure, and the residue was dried under high vacuum overnight to yield the acid **7** (5.80 g, 99%) as a white solid.

A mixture of acid **7** (5.57 g, 10.0 mmol) and amine salt **8** (3.10 g, 11.0 mmol) in anhydrous DMF (100 mL) was treated with DIEA (6.70 mL, 40.5 mmol) and HATU (5.70 g, 15.0 mmol). The resulting reaction mixture was stirred at room temperature for 2.5 h, then diluted with EtOAc (400 mL) and washed successively with aqueous 0.5 N HCl, saturated aqueous $NaHCO_3$, and saturated aqueous NaCl (250 mL each). The organic portion was dried (Na_2SO_4), filtered, and evaporated under reduced pressure. The residue was purified by flash column chromatography (RediSep Gold column, 2 × 80 g, gradient elution with 20–90% EtOAc/hexanes) to provide the bis-olefin compound **9** (6.50 g, 83%) as a white solid. **^{1}H NMR** (400 MHz, $CDCl_3$) δ 10.02 (s, 1 H), 7.81 (d, *J* = 8.8 Hz, 1 H), 7.18 (dd, *J* = 8.8, 2.8 Hz, 1 H), 7.13 (d, *J* = 2.8 Hz, 1 H), 7.11 (s, 1 H), 5.88 (br s, 1 H), 5.82–5.72 (m, 2 H), 5.42 (d, *J* = 9.2 Hz, 1 H), 5.26 (d, *J* = 17.2 Hz, 1 H), 5.14 (d, *J* = 11.6 Hz, 1 H), 5.00–4.90 (m, 2 H), 4.50 (t, *J* = 8.4 Hz, 1 H), 4.39–4.33 (m, 1 H), 4.18 (d, *J* = 11.6 Hz, 1 H), 4.02 (dd, *J* = 11.6, 4.0 Hz, 1 H), 3.93 (s, 3 H), 2.58–2.50 (m, 5 H), 2.10 (q, *J* = 8.4 Hz, 1 H), 2.05–1.98 (m, 3 H), 1.73–1.58 (m, 4 H), 1.49 (s, 3 H), 1.44–1.24 (m, 16 H),

0.92–0.86 (m, 1 H), 0.84–0.78 (m, 1 H); ^{13}**C NMR** (100 MHz, $CDCl_3$) δ 173.65, 172.52, 167.55, 160.31, 155.70, 155.16, 144.41, 140.87, 138.83, 134.33, 132.61, 128.96, 118.87, 118.54, 114.41, 105.96, 79.73, 74.59, 60.30, 55.67, 53.15, 52.37, 41.73, 36.56, 35.16, 34.25, 33.62, 32.24, 28.71, 28.67, 28.26, 25.31, 23.42, 19.84, 18.37, 14.27, 13.26 ppm; **HRMS** (ESI) *m/z*: $[M + H]^+$ calcd for $C_{39}H_{55}N_6O_9S$, 783.3746; found 783.3734.

***Tert*-Butyl ((2*R*,6*S*,13a*S*,14a*R*,16a*S*,*Z*)-2-((7-methoxy-3-methylquinoxalin-2-yl)oxy)-14a-(((1-methylcyclopropyl)sulfonyl)carbamoyl)-5,16-dioxo-1,2,3,5,6,7,8,9,10,11,13a,14,14a,15,16,16a-hexadecahydrocyclopropa[*e*]pyrrolo[1,2-*a*][1,4]diazacyclopentadecin-6-yl)carbamate (10).**

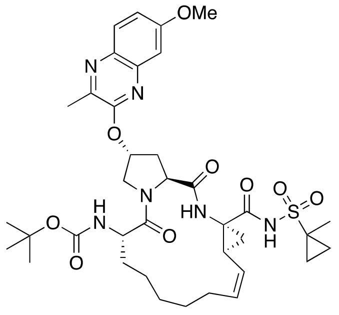

A degassed solution of bis-olefin **9** (6.20 g, 7.92 mmol) in 1,2-DCE (1600 mL) was heated to 50 °C under argon, then Zhan 1b catalyst (0.50 g, 0.68 mmol) was added in two portions over 10 min. The resulting reaction mixture was heated to 70 °C and stirred for 6 h. The reaction mixture was cooled to room temperature and solvents were evaporated under reduced pressure. The residue was purified by flash column chromatography (RediSep Gold column, 2 × 80 g, gradient elution with 20–90% EtOAc/hexanes) to yield the P1–P3 macrocyclic product **10** (4.20 g, 70%) as an off-white solid. ^{1}**H NMR** (400 MHz, $CDCl_3$) δ 10.16 (s, 1 H), 7.82 (d, *J* = 9.2 Hz, 1 H), 7.19–7.16 (m, 2 H), 6.92 (s, 1 H), 5.88 (br s, 1 H), 5.69 (q, *J* = 9.2 Hz, 1 H), 5.12 (d, *J* = 7.6 Hz, 1 H), 4.99 (t, *J* = 8.8 Hz, 1 H), 4.61 (t, *J* = 8.0 Hz, 1 H), 4.51 (d, *J* = 11.2 Hz, 1 H), 4.28–4.22 (m, 1 H), 4.03 (dd, *J* = 11.2, 4.0 Hz, 1 H), 3.95 (s, 3 H), 2.70–2.50 (m, 6 H), 2.31 (q, *J* = 8.8 Hz, 1 H), 1.92–1.66 (m, 4 H), 1.60–1.20 (m, 21 H), 0.85–0.78 (m, 2 H) ppm; ^{13}**C NMR** (100 MHz, $CDCl_3$) δ 177.16, 173.33, 166.94, 160.33, 155.32, 155.04, 144.46, 141.03, 134.20, 136.25, 128.66, 124.89, 118.93, 105.98, 79.85, 74.88, 59.46, 55.72, 53.08, 51.97, 44.73, 36.43, 34.61, 32.72, 29.65, 28.15, 27.06, 26.07, 22.21, 20.96, 19.71, 18.17, 14.51, 12.51 ppm; **HRMS** (ESI)

m/z: $[M + H]^+$ calcd for $C_{37}H_{51}N_6O_9S$, 755.3433; found 755.3404. Anal. HPLC: t_R 13.57 min, purity 99%.

(2*R*,6*S*,13a*S*,14a*R*,16a*S*,*Z*)-6-Amino-2-((7-methoxy-3-methylquinoxalin-2-yl)oxy)-*N*-((1-methylcyclopropyl)sulfonyl)-5,16-dioxo-1,2,3,6,7,8,9,10,11,13a,14,15,16,16a-tetradecahydrocyclopropa[*e*]pyrrolo[1,2-*a*][1,4]diazacyclopentadecine-14a(5*H*)-carboxamide hydrochloride (11).

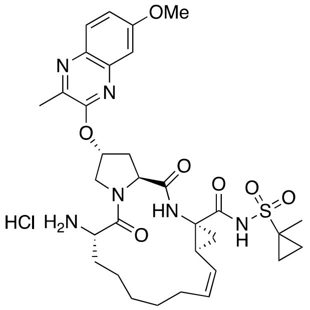

A solution of compound **10** (3.25 g, 4.31 mmol) in anhydrous CH_2CL_2 (15 mL) was treated with a solution of 4 N HCl in 1,4-dioxane (50 mL). The reaction mixture was stirred at room temperature for 3 h, concentrated under reduced pressure, and the residue was dried under high vacuum. The residue was triturated with diethyl ether (40 mL), and the solid was filtered, washed with Et_2O (2 × 15 mL), and dried under high vacuum to yield the amine salt **11** (2.90 g, 98%) as an off-white solid.

1-Methylcyclopropyl ((2*R*,6*S*,13a*S*,14a*R*,16a*S*,*Z*)-2-((7-methoxy-3-methylquinoxalin-2-yl)oxy)-14a-(((1-methylcyclopropyl)sulfonyl)carbamoyl)-5,16-di oxo-1,2,3,5,6,7,8,9,10,11,13a,14,14a,15,16,16a-hexadecahydrocyclopropa[*e*]pyrrolo [1,2-*a*][1,4]diazacyclopentadecin-6-yl)carbamate (P_4-1).

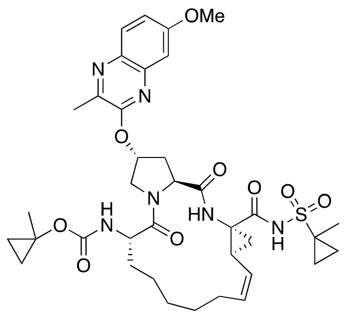

A solution of the amine salt **11** (0.2 g, 0.29 mmol) in anhydrous CH_3CN (10 mL) was treated with DIEA (0.354 mL, 2.02 mmol) and 1-methylcyclopropyl (4-nitrophenyl) carbonate (0.076 g, 0.32 mmol). The resulting reaction mixture was stirred at room temperature for 24 h, then concentrated under reduced pressure and dried under high vacuum. The residue was purified by flash column chromatography (RediSep Gold column, 24 g, gradient elution with 10–90% EtOAc/hexanes) to provide the target compound **P_4-1** (0.190 g, 83%) as a white solid. **^{1}H NMR** (500 MHz, $CDCl_3$) δ 10.16 (s, 1 H), 7.79 (d, *J* = 9.5 Hz, 1 H), 7.22–7.15 (m, 2 H), 7.03 (s, 1 H), 5.91 (br s, 1 H), 5.68 (q, *J* = 8.5 Hz, 1 H), 5.29 (d, *J* = 4.5 Hz, 1 H), 4.98 (t, *J* = 9.5 Hz, 1 H), 4.61 (t, *J* = 8.0 Hz, 1 H), 4.46 (d, *J* = 11.5 Hz, 1 H), 4.30 (t, *J* = 7.5 Hz, 1 H), 4.06 (dd, *J* = 11.5, 4.0 Hz, 1 H), 3.94 (s, 3 H), 2.57–2.50 (m, 6 H), 2.30 (q, *J* = 9.0 Hz, 1 H), 1.95–1.69 (m, 5 H), 1.62–1.52 (m, 1 H), 1.50–1.23 (m, 13 H), 0.86–0.78 (m, 2 H), 0.77–0.70 (m, 2 H), 0.55–0.48 (m, 2 H) ppm; **^{13}C NMR** (125 MHz, $CDCl_3$) δ 177.30, 173,01, 167.15, 160.43, 155.52, 155.45, 144.68, 141.14, 136.30, 128.90, 125.00, 118.97, 106.18, 74.88, 59.57, 56.76, 55.86, 53.18, 52.27, 44.79, 38.74, 36.60, 34.76, 32.75, 29.78, 27.31, 27.18, 26.25, 22.29, 21.47, 20.95, 19.93, 18.31, 14.62, 13.04, 12.90, 12.67 ppm; **HRMS** (ESI) *m/z*: $[M + H]^+$ calcd for $C_{37}H_{49}N_6O_9S$, 753.3276; found 753.3248.

1-Methylcyclobutyl ((2*R*,6*S*,13a*S*,14a*R*,16a*S*,*Z*)-2-((7-methoxy-3-methylquinoxalin-2-yl)oxy)-14a-(((1-methylcyclopropyl)sulfonyl)carbamoyl)-5,16-di oxo-1,2,3,5,6,7,8,9,10,11,13a,14,14a,15,16,16a-hexadecahydrocyclopropa[*e*]pyrrolo [1,2-*a*][1,4]diazacyclopentadecin-6-yl)carbamate (P_4-2).

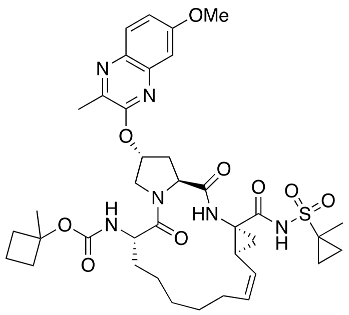

The same procedure was used as described above for compound **P_4-1**. A mixture of amine salt **11** (0.150 g, 0.22 mmol) was treated with DIEA (0.266 mL, 1.52 mmol) and 1-methylcyclobutyl (4-nitrophenyl) carbonate (0.060 g, 0.24 mmol) to provide the target compound **P_4-2** (0.140 g, 80%) as a white solid. **^{1}H NMR** (500 MHz, $CDCl_3$) δ 10.13 (s, 1 H), 7.80 (d, *J* = 9.5 Hz, 1 H), 7.20–7.16 (m, 2 H), 6.90 (s, 1 H), 5.89 (br s, 1 H), 5.70 (q, *J* = 8.5 Hz, 1 H), 5.21 (d, *J* = 7.5 Hz, 1 H), 5.00 (t, *J* = 9.5 Hz, 1 H), 4.61 (t, *J* = 7.5 Hz, 1 H), 4.46 (d, *J* = 11.5 Hz, 1 H), 4.28 (t, *J* = 7.5 Hz, 1 H), 4.04 (dd, *J* = 11.0, 3.5 Hz, 1 H), 3.95 (s, 3 H), 2.73–2.49 (m, 6 H), 2.31 (q, *J* = 9.0 Hz, 1 H), 2.15 (q, *J* = 9.0 Hz, 2 H), 1.99–1.77 (m, 6 H), 1.75–1.24 (m, 17 H), 0.85–0.79 (m, 2 H) ppm; **^{13}C NMR** (125 MHz, $CDCl_3$) δ 177.22, 173.30, 167.03, 160.40, 155.42, 154.61, 144.69, 141.10, 136.33, 134.42, 129.01, 125.04, 118.94, 106.16, 79.63, 74.88, 59.58, 55.86, 53.23, 52.14, 44.86, 38.75, 36.60, 35.39, 34.74, 32.91, 29.85, 27.29, 27.18, 26.22, 23.58, 22.37, 21.07, 20.00, 18.34, 14.65, 13.69, 12.71 ppm; **HRMS** (ESI) *m/z*: $[M + H]^+$ calcd for $C_{38}H_{51}N_6O_9S$, 767.3433; found 767.3406.

1-Methylcyclopentyl ((2*R*,6*S*,13a*S*,14a*R*,16a*S*,*Z*)-2-((7-methoxy-3-methylquinoxalin-2-yl)oxy)-14a-(((1-methylcyclopropyl)sulfonyl)carbamoyl)-5,16-dioxo-

1,2,3,5,6,7,8,9,10,11,13a,14,14a,15,16,16a-hexadecahydrocyclopropa[*e*]pyrrolo[1,2-*a*][1,4]diazacyclopentadecin-6-yl)carbamate (P4-4).

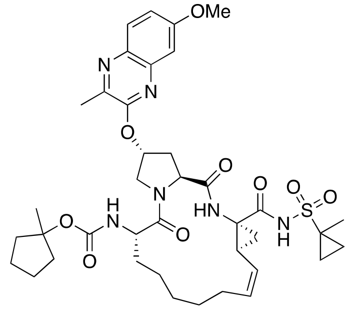

The same procedure was used as described above for compound **P4-1**. A mixture of amine salt **11** (0.25 g, 0.36 mmol) was treated with DIEA (0.45 mL, 2.58 mmol) and 1-methylcyclopentyl (4-nitrophenyl) carbonate (0.098 g, 0.37 mmol) to provide the target compound **P4-4** (0.24 g, 85%) as a white solid. **^{1}H NMR** (500 MHz, $CDCl_3$) δ 10.13 (s, 1 H), 7.79 (d, *J* = 10.0 Hz, 1 H), 7.18–-7.16 (m, 2 H), 6.87 (s, 1 H), 5.89 (br s, 1 H), 5.70 (q, *J* = 9.0 Hz, 1 H), 5.12 (d, *J* = 7.5 Hz, 1 H), 5.00 (t, *J* = 9.0 Hz, 1 H), 4.61 (t, *J* = 7.5 Hz, 1 H), 4.50 (d, *J* = 11.5 Hz, 1 H), 4.28 (t, *J* = 8.0 Hz, 1 H), 4.04 (dd, *J* = 11.0, 4.0 Hz, 1 H), 3.95 (s, 3 H), 2.69–2.50 (m, 6 H), 2.31 (q, *J* = 8.5 Hz, 1 H), 1.93–1.76 (m, 6 H), 1.65–1.25 (m, 21 H), 0.85–0.79 (m, 2 H) ppm; **^{13}C NMR** (125 MHz, $CDCl_3$) δ 177.11, 173.33, 166.90, 160.27, 155.31, 155.20, 144.50, 140.98, 136.24, 134.31, 128.89, 124.92, 118.78, 106.03, 89.54, 74.79, 59.47, 55.72, 53.11, 52.03, 44.75, 39.28, 39.08, 36.47, 34.61, 32.79, 29.71, 27.11, 27.06, 26.09, 24.59, 23.77, 22.26, 21.00, 19.86, 18.20, 14.52, 12.57 ppm; **HRMS** (ESI) *m/z*: $[M + H]^+$ calcd for $C_{39}H_{53}N_6O_9S^+$, 781.3589; found 781.3570.

1-Ethylcyclopentyl ((2*R*,6*S*,13a*S*,14a*R*,16a*S*, *Z*)-2-((7-methoxy-3-methylquinoxalin-2-yl) oxy)-14a-(((1-methylcyclopropyl) sulfonyl) carbamoyl)-5,16-dioxo-1,2,3,5,6,7,8,9,10,11,13a,14,14a,15,16,16a-hexadecahydrocyclopropa[*e*]pyrrolo[1,2-*a*][1,4] diazacyclopentadecin-6-yl) carbamate (P4-5).

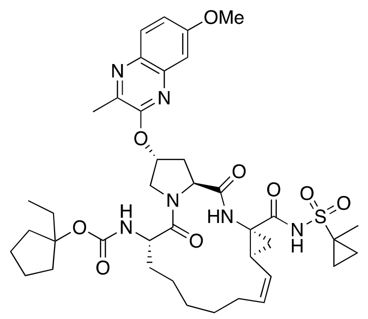

The same procedure was used as described above for compound **P4-1**. A mixture of amine salt **11** (0.120 g, 0.17 mmol) was treated with DIEA (0.210 mL, 1.21 mmol) and 1-ethylcyclopentyl (4-nitrophenyl) carbonate (0.050 g, 0.18 mmol) to provide the target compound **P4-5** (0.110 g, 76%) as a white solid. **^{1}H NMR** (500 MHz, $CDCl_3$) δ 10.12 (s, 1 H), 7.84 (d, *J* = 9.5 Hz, 1 H), 7.22–7.16 (m, 2 H), 6.86 (s, 1 H), 5.89 (br s, 1 H), 5.70 (q, *J* = 8.5 Hz, 1 H), 5.15 (d, *J* = 7.5 Hz, 1 H), 4.99 (t, *J* = 9.0 Hz, 1 H), 4.62 (t, *J* = 7.5 Hz, 1 H), 4.52 (d, *J* = 11.0 Hz, 1 H), 4.27 (t, *J* = 7.5 Hz, 1 H), 4.04 (dd, *J* = 11.5, 4.0 Hz, 1 H), 3.95 (s, 3 H), 2.74–2.52 (m, 6 H), 2.31 (q, *J* = 8.5 Hz, 1 H), 1.93–1.67 (m, 6 H), 1.63–1.23 (m, 20 H), 0.87–0.79 (m, 2 H), 0.76 (t, *J* = 7.5 Hz, 3 H) ppm; **^{13}C NMR** (125 MHz, $CDCl_3$) δ 177.20, 173.50, 166.98, 160.57, 155.48, 155.25, 144.50, 141.24, 136.37, 128.65, 125.06, 119.17, 106.18, 93.17, 75.09, 59.56, 55.88, 53.21, 52.18, 44.90, 38.76, 37.33, 37.32, 36.61, 34.74, 32.94, 30.25, 29.85, 27.20, 26.20, 24.14, 22.42, 21.13, 19.72, 18.34, 14.66, 12.71, 8.90 ppm; **HRMS** (ESI) *m/z*: $[M + H]^+$ calcd for $C_{40}H_{55}N_6O_9S$, 795.3746; found 795.3714.

Cyclohexyl ((2*R*,6*S*,13a*S*,14a*R*,16a*S*,*Z*)-2-((7-methoxy-3-methylquinoxalin-2-yl)oxy)-14a-(((1-methylcyclopropyl)sulfonyl)carbamoyl)-5,16-dioxo-1,2,3,5,6,7,8,9,10,11,13a,14,14a,15,16,16a-hexadecahydrocyclopropa[*e*]pyrrolo[1,2-*a*][1,4]diazacyclopentadecin-6-yl)carbamate (P4-6).

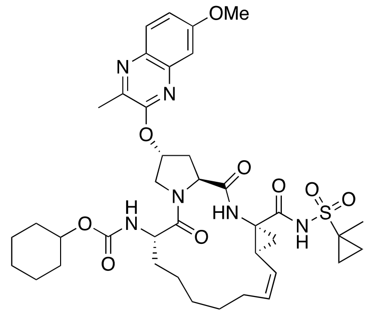

The same procedure was used as described above for compound **P4-1**. A mixture of amine salt **11** (0.100 g, 0.15 mmol) was treated with DIEA (0.2 mL, 1.02 mmol) and cyclohexyl (4-nitrophenyl) carbonate (0.043 g, 0.16 mmol) to provide the target compound **P4-6** (0.105 g, 88%) as a white solid. **^{1}H NMR** (500 MHz, $CDCl_3$) δ 10.13 (s, 1 H), 7.80 (d, *J* = 9.5 Hz, 1 H), 7.22–7.16 (m, 2 H), 6.86 (s, 1 H), 5.91 (br s, 1 H), 5.71 (q, *J* = 8.5 Hz, 1 H), 5.25 (d, *J* = 7.5 Hz, 1 H), 5.01 (t, *J* = 9.5 Hz, 1 H), 4.61 (t, *J* = 7.5 Hz, 1 H), 4.46 (d, *J* = 11.0 Hz, 1 H), 4.43–4.34 (m, 1 H), 4.30 (t, *J* = 7.5 Hz, 1 H), 4.05 (dd, *J* = 11.0, 4.0 Hz, 1 H), 3.95 (s, 3 H), 2.73–2.66 (m, 1 H), 2.64–2.49 (m, 5 H), 2.31 (q, *J* = 8.5 Hz, 1 H), 2.15 (q, *J* = 9.0 Hz, 2 H), 1.95–1.55 (m, 9 H), 1.54–1.22 (m, 15 H), 0.86–0.79 (m, 2 H) ppm; **^{13}C NMR** (125 MHz, $CDCl_3$) δ 177.26, 173.27, 166.98, 160.40, 155.59, 155.48, 144.81, 141.11, 136.32, 134.45, 129.04, 125.05, 118.91, 106.17, 74.84, 73.83, 59.64, 55.86, 53.18, 52.41, 44.89, 38.76, 36.60, 34.67, 32.74, 32.10, 32.04, 29.86, 27.28, 27.17, 26.20, 25.43, 23.94, 22.41, 21.12, 19.94, 18.35, 14.64, 12.72 ppm; **HRMS** (ESI) *m/z*: $[M + H]^+$ calcd for $C_{39}H_{53}N_6O_9S$, 781.3589; found 781.3565.

(1*R*,3*R*,5*S*)-bicyclo[3.1.0]hexan-3-yl ((2*R*,6*S*,13a*S*,14a*R*,16a*S*,*Z*)-2-((7-methoxy-3-methylquinoxalin-2-yl)oxy)-14a-(((1-methylcyclopropyl)sulfonyl)carbamoyl)-5,16-dioxo-1,2,3,5,6,7,8,9,10,11,13a,14,14a,15,16,16a-hexadecahydrocyclopropa[*e*]pyrrolo[1,2-*a*][1,4]diazacyclopentadecin-6-yl)carbamate (P4-7).

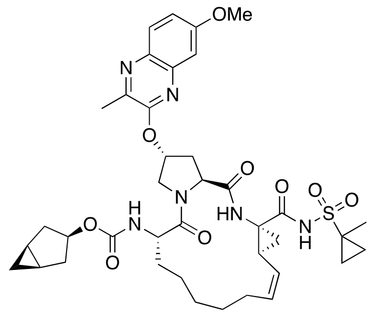

The same procedure was used as described above for compound **P4-1**. A mixture of amine salt **11** (0.120 g, 0.174 mmol) was treated with DIEA (0.226 mL, 1.22 mmol) and (1*R*,3*R*,5*S*)-bicyclo [3.1.0] hexan-3-yl (4-nitrophenyl) carbonate (0.050 g, 0.192 mmol) to provide the target compound **P4-7** (0.120 g, 85%) as a white solid. **^{1}H NMR** (500 MHz, $CDCl_3$) δ 10.11 (s, 1 H), 7.80 (d, *J* = 10.0 Hz, 1 H), 7.22–7.16 (m, 2 H), 6.83 (s, 1 H), 5.91 (br s, 1 H), 5.70 (q, *J* = 8.5 Hz, 1 H), 5.14 (d, *J* = 7.0 Hz, 1 H), 5.00 (t, *J* = 9.5 Hz, 1 H), 4.93 (t, *J* = 6.5 Hz, 1 H), 4.61 (t, *J* = 7.5 Hz, 1 H), 4.41 (d, *J* = 11.0 Hz, 1 H), 4.29 (t, *J* = 8.0 Hz, 1 H), 4.04 (dd, *J* = 11.0, 3.5 Hz, 1 H), 3.95 (s, 3 H), 2.77–2.53 (m, 1 H), 2.52–2.48 (m, 5 H), 2.30 (q, *J* = 9.0 Hz, 1 H), 2.10–1.72 (m, 6 H), 1.71–1.20 (m, 16 H), 0.87–0.79 (m, 2 H), 0.42 (dd, *J* = 12.5, 8.0 Hz, 1 H), 0.26 (d, *J* = 4.0 Hz, 1 H), ppm; **^{13}C NMR** (125 MHz, $CDCl_3$) δ 177.21, 173.16, 167.01, 160.40, 155.46, 155.43, 144.82, 141.10, 136.31, 134.47, 129.02, 125.04, 118.93, 106.18, 74.81, 59.63, 55.85, 53.21, 52.36, 44.85, 36.60, 35.80, 34.69, 32.76, 29.84, 27.32, 27.14, 26.20, 22.36, 21.16, 19.99, 18.35, 16.83, 14.62, 12.71, 10.57 ppm; **HRMS** (ESI) *m/z*: $[M + H]^+$ calcd for $C_{39}H_{51}N_6O_9S$, 779.3433; found 779.3411.

(2*R*,6*S*,13a*S*,14a*R*,16a*S*,*Z*)-6-((*S*)-2-Acetamido-3-methylbutanamido)-2-((7-methoxy-3-methylquinoxalin-2-yl)oxy)-*N*-((1-methylcyclopropyl)sulfonyl)-5,16-dioxo-1,2,3,6,7,8,9,10,11,13a,14,15,16,16a-tetradecahydrocyclopropa[*e*]pyrrolo[1,2-*a*][1,4]diazacyclopentadecine-14a(5*H*)-carboxamide (P_4P_5-1A).

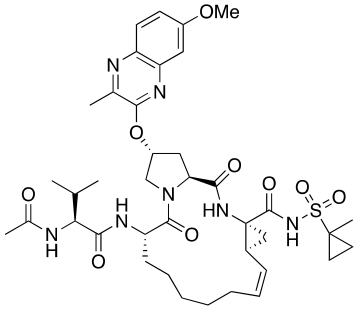

A mixture of amine salt **11** (0.25 g, 0.36 mmol) and *N*-Ac-Val-OH (0.075 g, 0.47 mmol) in anhydrous DMF (8 mL) was treated with DIEA (0.50 mL, 2.87 mmol) and HATU (0.30 g, 0.79 mmol). The resulting reaction mixture was stirred at room temperature for 5 h, then diluted with EtOAc (100 mL), and washed successively with aqueous 0.5 N HCl, saturated aqueous $NaHCO_3$, and saturated aqueous NaCl (75 mL each). The organic portion was dried (Na_2SO_4), filtered, and evaporated under reduced pressure. The residue was purified by flash column chromatography (RediSep Gold column, 12 g, gradient elution with 80–100% EtOAc/hexanes) to provide **P_4P_5-1A** (0.30 g, 80%) as a white solid. **^{1}H NMR** (500 MHz, CD_3OD) δ 7.76 (d, *J* = 9.0 Hz, 1 H), 7.26 (d, *J* = 3.0 Hz, 1 H), 7.20 (dd, *J* = 9.5, 3.0 Hz, 1 H), 5.98 (br s, 1 H), 5.69 (q, *J* = 9.0 Hz, 1 H), 5.05 (t, *J* = 9.0 Hz, 1 H), 4.68 (t, *J* = 9.0 Hz, 1 H), 4.60 (d, *J* = 12.0 Hz, 1 H), 4.42 (dd, *J* = 11.5, 3.0 Hz, 1 H), 4.09 (dd, *J* = 11.5, 4.0, Hz, 1 H), 4.05 (d, *J* = 7.5 Hz, 1 H), 3.93 (s, 3 H), 2.72–2.62 (m, 2 H), 2.58–2.52 (m, 1 H), 2.50 (s, 3 H), 2.43 (q, *J* = 8.5 Hz, 1 H), 1.97–1.81 (m, 6 H), 1.72 (dd, *J* = 8.5, 5.5 Hz, 1 H), 1.64–1.29 (m, 14 H), 0.90–0.84 (m, 2 H), 0.79 (t, *J* = 7.5 Hz, 6 H) ppm; **^{13}C NMR** (125 MHz, CD_3OD) δ 179.28, 173.79, 173.16, 172.76, 169.47, 162.01, 157.02, 146.26, 142.65, 136.85, 134.82, 129.24, 126.43, 119.75, 107.33, 76.70, 60.78, 59.49, 56.26, 54.41, 52.42, 45.29, 37.59, 35.96, 33.29, 32.07, 31.00, 28.60, 28.35, 27.82, 23.21, 22.37, 21.78, 19.80, 19.71, 18.68, 18.43, 14.57, 13.02 ppm; **HRMS** (ESI) *m/z*: $[M + H]^+$ calcd for $C_{39}H_{54}N_7O_9S^+$, 796.3698; found 796.3679.

(2*R*,6*S*,13a*S*,14a*R*,16a*S*,*Z*)-6-((*S*)-2-Acetamido-3,3-dimethylbutanamido)-2-((7-methoxy-3-methylquinoxalin-2-yl)oxy)-*N*-((1-methylcyclopropyl)sulfonyl)-5,16-dioxo-1,2,3,6,7,8,9,10,11,13a,14,15,16,16a-tetradecahydrocyclopropa[*e*]pyrrolo[1,2-*a*][1,4]diazacyclopentadecine-14a(5*H*)-carboxamide (P_4P_5-2A).

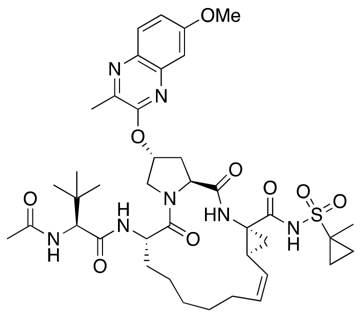

The same procedure was used as described above for compound **P_4P_5-1A**. A mixture of amine salt **11** (0.31 g, 0.45 mmol) and *N*-Ac-Tle-OH (0.10 g, 0.58 mmol) was treated with DIEA (0.60 mL, 3.50 mmol)) and HATU (0.35 g, 0.92 mmol) to provide the target compound **P_4P_5-2A** (0.28 g, 77%) as a white solid. **^{1}H NMR** (500 MHz, CD_3OD) δ 7.76 (d, *J* = 9.0 Hz, 1 H), 7.26 (d, *J* = 2.5 Hz, 1 H), 7.21 (dd, *J* = 9.0, 3.0 Hz, 1 H), 5.95 (br s, 1 H), 5.70 (q, *J* = 8.5 Hz, 1 H), 5.05 (t, *J* = 9.0 Hz, 1 H), 4.68 (dd, *J* = 9.0, 7.5 Hz, 1 H), 4.63 (d, *J* = 11.5 Hz, 1 H), 4.44 (dd, *J* = 11.5, 3.0 Hz, 1 H), 4.13–4.09 (m, 2 H), 3.94 (s, 3 H), 2.73–2.63 (m, 2 H), 2.57–2.46 (m, 5 H), 2.00–1.93 (m, 1 H), 1.91–1.83 (m, 4 H), 1.72 (dd, *J* = 8.5, 5.5 Hz, 1 H), 1.64–1.36 (m, 13 H), 1.33–1.26 (m, 1 H), 0.90–0.84 (m, 2 H), 0.74 (s, 9 H) ppm; **^{13}C NMR** (125 MHz, CD_3OD) δ 179.38, 173.54, 172.91, 171.70, 169.52, 162.05, 156.98, 146.05, 142.68, 136.89, 134.86, 129.27, 126.45, 119.79, 107.34, 76.88, 61.43, 60.87, 56.26, 54.47, 52.16, 45.36, 37.59, 36.09, 35.30, 33.27, 30.92, 28.65, 28.23, 27.87, 27.04, 23.29, 22.37, 21.75, 19.91, 18.43, 14.57, 13.01 ppm; **HRMS** (ESI) *m/z*: [M + H]$^+$ calcd for $C_{40}H_{56}N_7O_9S^+$, 810.3855; found 810.3836.

(2*R*,6*S*,13a*S*,14a*R*,16a*S*, *Z*)-6-((*S*)-2-acetamido-2-cyclopentylacetamido)-2-((7-methoxy-3-methylquinoxalin-2-yl)oxy)-*N*-((1-methylcyclopropyl)sulfonyl)-5,16-dioxo-1,2,3,6,7,8,9,10,11,13a,14,15,16,16a-tetradecahydrocyclopropa[*e*]pyrrolo[1,2-*a*][1,4]diazacyclopentadecine-14a(5*H*)-carboxamide (P_4P_5-3A).

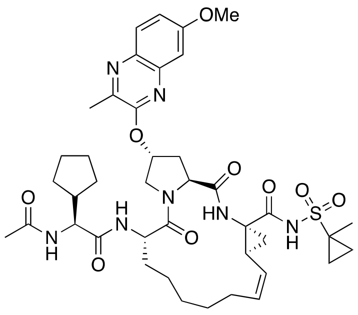

The same procedure was used as described above for compound **P_4P_5-1A**. A mixture of amine salt **11** (0.25 g, 0.36 mmol) and *N*-Ac-cyclopentylglycine (0.075 g, 0.47 mmol) was treated with DIEA (0.50 mL, 2.87 mmol) and HATU (0.30 g, 0.79 mmol) to provide the target compound **P_4P_5-3A** (0.30 g, 80%) as a white solid. **^{1}H NMR** (500 MHz, CD_3OD) δ 7.76 (d, *J* = 9.5 Hz, 1 H), 7.27 (d, *J* = 3.0 Hz, 1 H), 7.21 (dd, *J* = 9.0, 3.0 Hz, 1 H), 5.99 (br s, 1 H), 5.70 (q, *J* = 8.5 Hz, 1 H), 5.05 (t, *J* = 9.0 Hz, 1 H), 4.66 (t, *J* = 8.5 Hz, 1 H), 4.61 (d, *J* = 11.5 Hz, 1 H), 4.40 (dd, *J* = 11.0, 2.5 Hz, 1 H), 4.10 (dd, *J* = 11.5, 3.5, Hz, 1 H), 4.03 (d, *J* = 9.0 Hz, 1 H), 3.94 (s, 3 H), 2.70–2.62 (m, 2 H), 2.58–2.51 (m, 4 H), 2.44 (q, *J* = 9.0 Hz, 1 H), 1.99–1.85 (m, 6 H), 1.73 (dd, *J* = 8.0, 5.5 Hz, 1 H), 1.68–1.16 (m, 22 H), 0.90–0.84 (m, 2 H) ppm; **^{13}C NMR** (125 MHz, CD_3OD) δ 179.36, 173.69, 173.28, 173.01, 169.51, 162.04, 157.06, 146.26, 142.70, 136.84, 134.84, 129.25, 126.46, 119.76, 107.35, 76.72, 60.76, 58.21, 56.27, 54.37, 52.44, 45.32, 43.48, 37.60, 35.98, 33.18, 31.02, 30.19, 30.17, 28.62, 28.31, 27.83, 26.14, 25.77, 23.24, 22.30, 21.74, 19.77, 18.44, 14.57, 13.01 ppm; **HRMS** (ESI) *m/z*: $[M + H]^+$ calcd for $C_{41}H_{56}N_7O_9S^+$, 822.3855; found 822.3836.

(2*R*,6*S*,13a*S*,14a*R*,16a*S*,*Z*)-6-((2*S*,3*S*)-2-acetamido-3-methylpentanamido)-2-((7-methoxy-3-methylquinoxalin-2-yl)oxy)-*N*-((1-methylcyclopropyl)sulfonyl)-5,16-dioxo-

1,2,3,6,7,8,9,10,11,13a,14,15,16,16a-tetradecahydrocyclopropa[*e*]pyrrolo[1,2-*a*][1,4]diazacyclopentadecine-14a(5*H*)-carboxamide (P_4P_5-4).

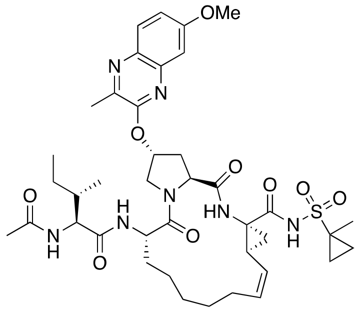

The same procedure was used as described above for compound **P_4P_5-1A**. A mixture of amine salt **11** (0.15 g, 0.22 mmol) and *N*-Ac-Ile-OH (0.061 g, 0.35 mmol) was treated with DIEA (0.23 mL, 1.4 mmol) and HATU (0.21 g, 0.55 mmol) to provide the target compound **P_4P_5-4** (0.10 g, 56%) as a white solid. **^{1}H NMR** (500 MHz, CD_3OD) δ 8.15 (d, *J* = 7.0 Hz, 1 H), 7.77–7.73 (m, 2 H), 7.25 (d, *J* = 2.5 Hz, 1 H), 7.20 (dd, *J* = 9.0, 2.5 Hz, 1 H), 5.98 (br s, 1 H), 5.70 (q, *J* = 8.5 Hz, 1 H), 5.05 (t, *J* = 9.0 Hz, 1 H), 4.67 (t, *J* = 8.5 Hz, 1 H), 4.62 (d, *J* = 12.0 Hz, 1 H), 4.44–4.40 (m, 1 H), 4.11–4.05 (m, 2 H), 3.93 (s, 3 H), 2.71–2.62 (m, 2 H), 2.58–2.50 (m, 4 H), 2.43 (q, *J* = 8.5 Hz, 1 H), 1.99–1.84 (m, 5 H), 1.73 (dd, *J* = 8.0, 5.5 Hz, 1 H), 1.64–1.25 (m, 15 H), 1.09–1.00 (m, 1 H), 0.90–0.84 (m, 2 H), 0.82 (t, *J* = 7.5 Hz, 3 H), 0.73 (d, *J* = 7.0 Hz, 3 H) ppm; **^{13}C NMR** (125 MHz, CD_3OD) δ 179.32, 173.67, 173.04, 172.90, 169.48, 162.00, 157.02, 146.25, 142.65, 136.84, 134.81, 129.23, 126.43, 119.74, 107.33, 76.71, 60.75, 58.56, 56.27, 54.38, 52.43, 45.31, 38.16, 37.59, 35.97, 33.21, 31.01, 28.61, 28.32, 27.81, 25.96, 23.23, 22.36, 21.75, 19.80, 18.44, 15.87, 14.57, 13.01, 11.29 ppm; **HRMS** (ESI) *m/z*: $[M + H]^+$ calcd for $C_{40}H_{56}N_7O_9S^+$, 810.3855; found 810.3832.

Methyl ((*S*)-1-(((2*R*,6*S*,13a*S*,14a*R*,16a*S*,*Z*)-2-((7-methoxy-3-methylquinoxalin-2-yl)oxy)-14a-(((1-methylcyclopropyl)sulfonyl)carbamoyl)-5,16-dioxo-1,2,3,5,6,7,8,9,10,11,13a,14,14a,15,16,16a-hexadecahydrocyclopropa[*e*]pyrrolo[1,2-*a*][1,4]diazacyclopentadecin-6-yl)amino)-3-methyl-1-oxobutan-2-yl)carbamate (P_4P_5-1B).

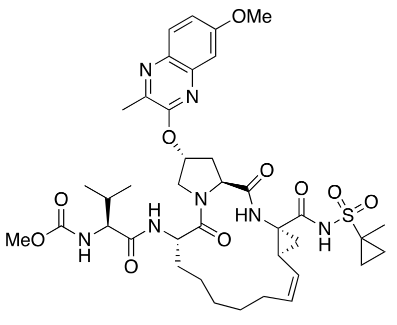

The same procedure was used as described above for compound **P_4P_5-1A**. A mixture of amine salt **11** (0.25 g, 0.36 mmol) and methoxycarbonyl-*L*-valine (0.085 g, 0.48 mmol) was treated with DIEA (0.50 mL, 2.87 mmol) and HATU (0.30 g, 0.79 mmol) to provide the target compound **P_4P_5-1B** (0.24 g, 82%) as a white solid. **^{1}H NMR** (500 MHz, $CDCl_3$) δ 10.25 (s, 1 H), 8.71 (br s, 1 H), 8.43 (br s, 1 H), 7.80 (d, *J* = 9.5 Hz, 1 H), 7.21–7.18 (m, 2 H), 5.95 (br s, 1 H), 5.72 (q, *J* = 8.5 Hz, 1 H), 5.48 (d, *J* = 9.5 Hz, 1 H), 4.98–4.89 (m, 2 H), 4.67 (t, *J* = 8.0 Hz, 1 H), 4.45 (d, *J* = 11.5 Hz, 1 H), 4.25 (dd, *J* = 9.5, 4.5 Hz, 1 H), 4.19 (dd, *J* = 11.5, 4.0 Hz, 1 H), 3.95 (s, 3 H), 3.64 (s, 3 H), 2.80–2.65 (m, 3 H), 2.43 (s, 3 H), 2.21 (q, *J* = 8.5 Hz, 1 H), 2.14–2.07 (m, 1 H), 1.87–1.65 (m, 3 H), 1.58–1.36 (m, 10 H), 1.31–1.17 (m, 2 H), 1.01 (dd, *J* = 9.0, 5.5 Hz, 1 H), 0.82–0.77 (m, 5 H), 0.67 (d, *J* = 7.0 Hz, 3 H) ppm; **^{13}C NMR** (125 MHz, $CDCl_3$) δ 177.09, 173.68, 170.18, 167.08, 160.67, 157.98, 155.22, 143.79, 141.16, 136.88, 134.23, 128.91, 124.87, 119.39, 106.12, 75.07, 59.56, 58.46, 55.90, 54.14, 53.30, 50.23, 44.37, 36.61, 35.53, 34.69, 32.78, 28.66, 27.71, 27.51, 26.90, 22.39, 20.06, 19.72, 19.61, 18.35, 16.70, 14.72, 12.72 ppm; **HRMS** (ESI) *m/z*: $[M + H]^+$ calcd for $C_{39}H_{54}N_7O_{10}S^+$, 812.3647; found 812.3624.

Methyl ((*S*)-1-(((2*R*,6*S*,13a*S*,14a*R*,16a*S*,*Z*)-2-((7-methoxy-3-methylquinoxalin-2-yl)oxy)-14a-(((1-methylcyclopropyl)sulfonyl)carbamoyl)-5,16-dioxo-

1,2,3,5,6,7,8,9,10,11,13a,14,14a,15,16,16a-hexadecahydrocyclopropa[*e*]pyrrolo[1,2-*a*][1,4]diazacyclopentadecin-6-yl)amino)-3,3-dimethyl-1-oxobutan-2-yl)carbamate (P_4P_5-2B).

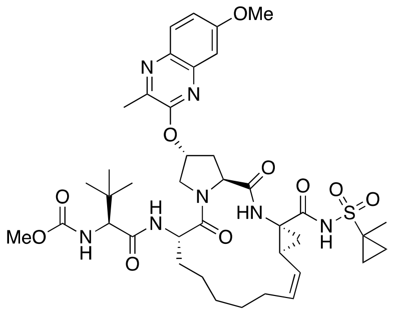

The same procedure was used as described above for compound **P_4P_5-1A**. A mixture of amine salt **11** (0.25 g, 0.36 mmol) and methoxycarbonyl-*L*-*tert*-leucine (0.093 g, 0.48 mmol) was treated with DIEA (0.50 mL, 2.87 mmol) and HATU (0.30 g, 0.79 mmol) to provide the target compound **P_4P_5-2B** (0.25 g, 84%) as a white solid. **^{1}H NMR** (500 MHz, $CDCl_3$) δ 10.22 (s, 1 H), 8.64 (br s, 1 H), 8.04 (d, *J* = 8.5 Hz, 1 H), 7.77 (d, *J* = 9.0 Hz, 1 H), 7.20–7.16 (m, 2 H), 5.89 (br s, 1 H), 5.68 (q, *J* = 8.5 Hz, 1 H), 5.48 (d, *J* = 10.0 Hz, 1 H), 4.96–4.91 (m, 2 H), 4.70 (t, *J* = 8.0 Hz, 1 H), 4.43 (d, *J* = 12.0 Hz, 1 H), 4.19 (dd, *J* = 11.5, 4.0 Hz, 1 H), 3.95 (s, 3 H), 3.86 (d, *J* = 10.0 Hz, 1 H), 3.63 (s, 3 H), 2.78–2.63 (m, 3 H), 2.45 (s, 3 H), 2.10 (q, *J* = 8.5 Hz, 2 H), 1.85–1.70 (m, 3 H), 1.50–1.13 (m, 11 H), 0.98 (dd, *J* = 10.4, 6.0 Hz, 1 H), 0.83–0.77 (m, 2 H), 0.65 (s, 9 H) ppm; **^{13}C NMR** (125 MHz, $CDCl_3$) δ 176.65, 172.94, 169.36, 166.94, 160.56, 157.69, 155.14, 143.75, 141.13, 136.92, 134.45, 128.99, 124.90, 119.23, 106.12, 75.10, 62.27, 59.25, 55.87, 54.13, 53.24, 50.35, 44.09, 36.64, 35.40, 35.10, 34.38, 28.74, 27.56, 27.50, 26.73, 26.52, 22.89, 19.92, 19.80, 18.31, 14.57, 12.73 ppm; **HRMS** (ESI) *m/z*: $[M + H]^+$ calcd for $C_{40}H_{56}N_7O_{10}S^+$, 826.3804; found 826.3778.

Methyl ((*S*)-1-cyclopentyl-2-(((2*R*,6*S*,13a*S*,14a*R*,16a*S*,*Z*)-2-((7-methoxy-3-methylquinoxalin-2-yl)oxy)-14a-(((1-methylcyclopropyl)sulfonyl)carbamoyl)-5,16-dioxo-1,2,3,5,6,7,8,9,10,11,13a,14,14a,15,16,16a-hexadecahydrocyclopropa[*e*]pyrrolo[1,2-*a*][1,4]diazacyclopentadecin-6-yl)amino)-2-oxoethyl)carbamate (P_4P_5-3B).

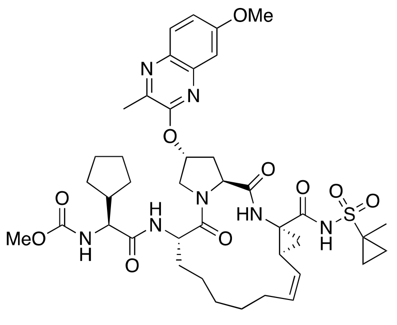

The same procedure was used as described above for compound **P_4P_5-1A**. A mixture of amine salt **11** (0.25 g, 0.36 mmol) and methoxycarbonyl-*L*-cyclopentylglycine (0.096 g, 0.48 mmol) was treated with DIEA (0.50 mL, 2.87 mmol) and HATU (0.30 g, 0.79 mmol) to provide the target compound **P_4P_5-3B** (0.25 g, 83%) as a white solid. **^{1}H NMR** (500 MHz, $CDCl_3$) δ 10.26 (s, 1 H), 8.73 (br s, 1 H), 8.40 (br s, 1 H), 7.79 (d, *J* = 10.0 Hz, 1 H), 7.20–7.18 (m, 2 H), 5.96 (br s, 1 H), 5.71 (q, *J* = 9.0 Hz, 1 H), 5.52 (d, *J* = 9.5 Hz, 1 H), 4.97–4.89 (m, 2 H), 4.68 (t, *J* = 8.0 Hz, 1 H), 4.43 (d, *J* = 11.5 Hz, 1 H), 4.37 (dd, *J* = 9.5, 5.0 Hz, 1 H), 4.21 (dd, *J* = 11.5, 4.5 Hz, 1 H), 3.95 (s, 3 H), 3.64 (s, 3 H), 2.72–2.67 (m, 2 H), 2.47 (s, 3 H), 2.17 (q, *J* = 8.5 Hz, 1 H), 2.09–2.04 (m, 1 H), 1.96–1.74 (m, 4 H), 1.54–1.32 (m, 20 H), 1.03 (dd, *J* = 9.0, 6.0 Hz, 1 H), 0.84–0.79 (m, 2 H) ppm; **^{13}C NMR** (125 MHz, $CDCl_3$) δ 177.12, 173.43, 170.61, 167.07, 160.58, 157.94, 155.27, 143.87, 141.14, 136.86, 134.47, 129.03, 124.86, 119.25, 106.14, 75.04, 59.37, 56.26, 55.90, 54.03, 53.26, 50.27, 44.28, 43.56, 36.61, 35.45, 34.67, 29.39, 28.74, 27.65, 27.51, 26.80, 25.57, 25.37, 22.41, 19.65, 18.33, 14.68, 12.71 ppm; **HRMS** (ESI) *m/z*: $[M + H]^+$ calcd for $C_{41}H_{56}N_7O_{10}S^+$, 838.3804; found 838.3774.

Methyl (1-(((2*R*,6*S*,13a*S*,14a*R*,16a*S*,*Z*)-2-((7-methoxy-3-methylquinoxalin-2-yl)oxy)-14a-(((1-methylcyclopropyl)sulfonyl)carbamoyl)-5,16-dioxo-1,2,3,5,6,7,8,9,10,11,13a,14,14a,15,16,16a-hexadecahydrocyclopropa[*e*]pyrrolo[1,2-*a*][1,4]diazacyclopentadecin-6-yl)carbamoyl)cyclopentyl)carbamate (P_4P_5-5).

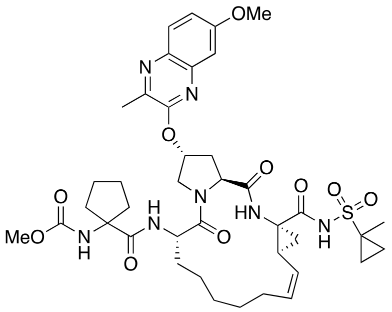

The same procedure was used as described above for compound **P_4P_5-1A**. A mixture of amine salt **11** (0.25 g, 0.36 mmol) and methoxycarbonyl-*L*-cycloleucine (0.089 g, 0.48 mmol) was treated with DIEA (0.50 mL, 2.87 mmol) and HATU (0.30 g, 0.79 mmol) to provide the target compound **P_4P_5-5** (0.20 g, 67%) as a white solid. **^{1}H NMR** (500 MHz, $CDCl_3$) δ 10.41 (s, 1 H), 8.08 (br s, 1 H), 7.77 (d, J = 9.0 Hz, 1 H), 7.18–7.15 (m, 2 H), 6.86 (d, J = 7.0 Hz, 1 H), 5.96 (br s, 1 H), 5.71 (q, J = 9.0 Hz, 1 H), 5.50 (br s, 1 H), 5.00 (t, J = 9.5 Hz, 1 H), 4.62 (t, J = 7.5 Hz, 1 H), 4.53 (br s, 1 H), 4.36 (d, J = 11.5 Hz, 1 H), 4.14 (dd, J = 11.0, 4.5 Hz, 1 H), 3.93 (s, 3 H), 3.61 (s, 3 H), 2.75–2.69 (m, 1 H), 2.62–2.55 (m, 1 H), 2.51–2.46 (m, 4 H), 2.14 (q, J = 8.5 Hz, 2 H), 1.96–1.45 (m, 18 H), 1.40–1.23 (m, 5 H), 0.86–0.81 (m, 2 H) ppm; **^{13}C NMR** (125 MHz, $CDCl_3$) δ 176.93, 173.94, 173.00, 167.34, 160.42, 156.24, 155.38, 144.41, 141.11, 136.43, 134.46, 129.00, 125.24, 119.00, 106.17, 74.75, 67.04, 59.33, 55.86, 52.96, 52.32, 51.64, 44.48, 38.73, 37.91, 36.61, 36.18, 34.62, 32.23, 29.81, 27.82, 27.22, 26.04, 24.22, 22.67, 19.93, 18.24, 14.76, 12.63 ppm; **HRMS** (ESI) *m/z*: $[M + H]^+$ calcd for $C_{40}H_{54}N_7O_{10}S^+$, 824.3647; found 824.3618.

Methyl ((*S*)-1-cyclohexyl-2-(((2*R*,6*S*,13a*S*,14a*R*,16a*S*,*Z*)-2-((7-methoxy-3-methylquinoxalin-2-yl)oxy)-14a-(((1-methylcyclopropyl)sulfonyl)carbamoyl)-5,16-dioxo-1,2,3,5,6,7,8,9,10,11,13a,14,14a,15,16,16a-hexadecahydrocyclopropa[*e*]pyrrolo[1,2-*a*][1,4]diazacyclopentadecin-6-yl)amino)-2-oxoethyl)carbamate (P_4P_5-6).

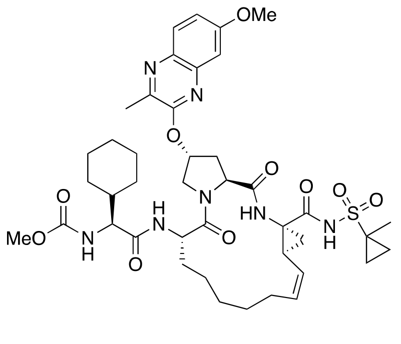

The same procedure was used as described above for compound **P_4P_5-1A**. A mixture of amine salt **11** (0.120 g, 0.170 mmol) and methoxycarbonyl-*L*-cyclohexylglycine (0.057 g, 0.26 mmol) was treated with DIEA (0.253 mL, 1.36 mmol) and HATU (0.143 g, 0.374 mmol) to provide the target compound **P_4P_5-6** (0.132 g, 84%) as a white solid. **^{1}H NMR** (500 MHz, $CDCl_3$) δ 10.21 (s, 1 H), 8.32 (br s, 1 H), 7.91 (br s, 1 H), 7.80(d, J = 9.5 Hz, 1 H), 7.21–7.18 (m, 2 H), 5.96 (br s, 1 H), 5.66 (q, J = 9.0 Hz, 1 H), 5.35 (d, J = 9.5 Hz, 1 H), 4.95 (t, J = 8.5 Hz, 1 H), 4.84 (t, J = 8.5 Hz, 1 H), 4.72 (t, J = 7.5 Hz, 1 H), 4.45 (d, J = 11.0 Hz, 1 H), 4.19 (dd, J = 11.5, 4.0 Hz, 1 H), 4.11 (dd, J = 9.0, 6.0 Hz, 1 H), 3.96 (s, 3 H), 3.65 (s, 3 H), 2.76–2.62 (m, 3 H), 2.49 (s, 3 H), 2.12 (q, J = 8.5 Hz, 1 H), 2.05–1.95 (m, 1 H), 1.86–1.74 (m, 3 H), 1.70–1.34 (m, 16 H), 1.27–0.79 (m, 9 H) ppm; **^{13}C NMR** (125 MHz, $CDCl_3$) δ 177.01, 173.07, 170.57, 166.98, 160.55, 157.60, 155.31, 144.15, 141.18, 136.96, 134.49, 129.06, 124.86, 119.17, 106.17, 74.91, 59.19, 59.10, 55.91, 53.90, 53.03, 50.78, 44.36, 41.54, 38.75, 36.65, 35.19, 34.32, 29.88, 29.19, 27.90, 27.47, 27.41, 26.45, 26.07, 26.01, 25.86, 22.62, 20.06, 19.89, 18.33, 14.63, 12.77 ppm; HRMS (ESI) *m/z*: $[M + H]^+$ calcd for $C_{42}H_{58}N_7O_{10}S$, 852.3960; found 852.3935.
