# Supplementary material for: Avoiding Drug Resistance by Substrate Envelope-Guided Design: Toward Potent and Robust HCV NS3/4A Protease Inhibitors
Source: mBio. 2020 Mar 31;11(2):e00172-20. doi: 10.1128/mBio.00172-20 (PMC7157764; doi:10.1128/mBio.00172-20)
Supplement: SCHEME S1 [file mBio.00172-20-ss001.pdf]

Scheme S1. Synthesis of HCV NS3/4A protease inhibitors

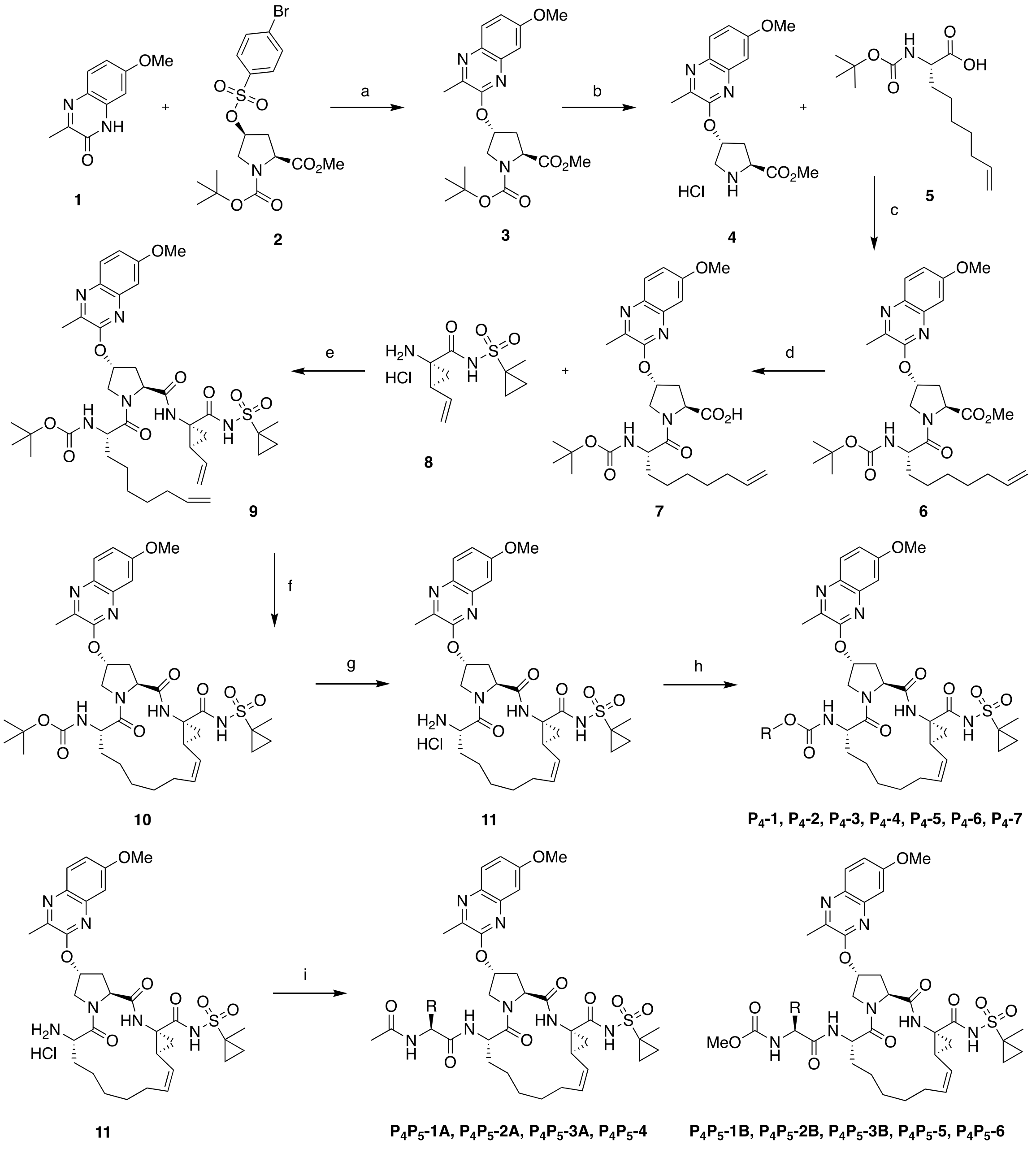

Reagents and Conditions: (a) Cs_2CO_3, NMP, 55 °C, 6 h; (b) 4 N HCl in dioxane, CH_2Cl_2, RT, 3 h; (c) HATU, DIEA, DMF, RT, 4 h; (d) $LiOH.H_2O$, THF, H_2O, RT, 24 h; (e) HATU, DIEA, DMF, RT, 2 h; (f) Zhan 1b catalyst, 1,2-DCE, 70 °C, 6 h; (g) 4 N HCl in dioxane, RT, 3 h; (h) alcohol-(4-nitrophenyl) carbonate, DIEA, CH_3CN, RT, 36 h; (i) *N*-protected amino acid, HATU, DIEA, DMF, RT, 4 h.
